# Under-Expression of Chemosensory Genes in Domiciliary Bugs of the Chagas Disease Vector *Triatoma brasiliensis*

**DOI:** 10.1371/journal.pntd.0005067

**Published:** 2016-10-28

**Authors:** Axelle Marchant, Florence Mougel, Emmanuelle Jacquin-Joly, Jane Costa, Carlos Eduardo Almeida, Myriam Harry

**Affiliations:** 1 UMR Evolution, Génomes, Comportement, Ecologie, CNRS-IRD- Univ. Paris-Sud, Université Paris Saclay, Campus CNRS, Gif-sur-Yvette – France; 2 UFR Sciences, Université Paris Sud, Orsay, France; 3 INRA, UMR 1392, Institut d’Ecologie et des Sciences de l’Environnement de Paris, Route de Saint Cyr, Versailles, France; 4 Laboratório de Biodiversidade Entomológica; Instituto Oswaldo Cruz - Fiocruz; Rio de Janeiro; Brasil Instituto Oswaldo Cruz, Fiocruz – Brazil; 5 Universidade Estadual de Campinas (Uncamp), Campinas São Paulo – Brazil; 6 Universidade Federal da Paraíba (UFPB), Paraíba – Brazil; New York University School of Medicine, UNITED STATES

## Abstract

**Background:**

In Latin America, the bloodsucking bugs Triatominae are vectors of *Trypanosoma cruzi*, the parasite that causes Chagas disease. Chemical elimination programs have been launched to control Chagas disease vectors. However, the disease persists because native vectors from sylvatic habitats are able to (re)colonize houses—a process called domiciliation. *Triatoma brasiliensis* is one example. Because the chemosensory system allows insects to interact with their environment and plays a key role in insect adaption, we conducted a descriptive and comparative study of the chemosensory transcriptome of *T*. *brasiliensis* samples from different ecotopes.

**Methodology/Principal Finding:**

In a reference transcriptome built using *de novo* assembly, we found transcripts encoding 27 odorant-binding proteins (OBPs), 17 chemosensory proteins (CSPs), 3 odorant receptors (ORs), 5 transient receptor potential channel (TRPs), 1 sensory neuron membrane protein (SNMPs), 25 takeout proteins, 72 cytochrome P450s, 5 gluthatione S-transferases, and 49 cuticular proteins. Using protein phylogenies, we showed that most of the OBPs and CSPs for *T*. *brasiliensis* had well supported orthologs in the kissing bug *Rhodnius prolixus*. We also showed a higher number of these genes within the bloodsucking bugs and more generally within all Hemipterans compared to the other species in the super-order Paraneoptera. Using both DESeq2 and EdgeR software, we performed differential expression analyses between samples of *T*. *brasiliensis*, taking into account their environment (sylvatic, peridomiciliary and domiciliary) and sex. We also searched clusters of co-expressed contigs using HTSCluster. Among differentially expressed (DE) contigs, most were under-expressed in the chemosensory organs of the domiciliary bugs compared to the other samples and in females compared to males. We clearly identified DE genes that play a role in the chemosensory system.

**Conclusion/Significance:**

Chemosensory genes could be good candidates for genes that contribute to adaptation or plastic rearrangement to an anthropogenic system. The domiciliary environment probably includes less diversity of xenobiotics and probably has more stable abiotic parameters than do sylvatic and peridomiciliary environments. This could explain why both detoxification and cuticle protein genes are less expressed in domiciliary bugs. Understanding the molecular basis for how vectors adapt to human dwellings may reveal new tools to control disease vectors; for example, by disrupting chemical communication.

## Author Summary

In Latin America, bloodsucking bugs are vectors of *Trypanosoma cruzi*, the parasite that causes Chagas disease, which is one of the most important public health problems for rural human populations. Though chemical control campaigns have been effective against vectors, the disease persists because native vectors from natural habitats have been able to recolonize human habitations. This is the case of *Triatoma brasiliensis*. Its capacity to adapt to a new habitat could be linked to changes in the number and/or the expression of chemosensory system genes, particularly those encoding odorant-binding proteins (OBPs) and chemosensory proteins (CSPs), which are important for detecting odor stimuli. This study looks at the chemosensory system of Triatominae in an attempt to document the adaptation process and the domiciliation of disease vectors. We used RNAseq to annotate chemosensory genes and to evidence differential gene expression in *T*. *brasiliensis* samples from different habitats.

## Introduction

Chagas disease is a potentially fatal parasitic disease caused by *Trypanosoma cruzi*, an endemic kinetoplastida that has infected five million people in Latin America [1] and is transmitted by blood-sucking bugs (Hemiptera, Reduviidae, Triatominae). Chemical control campaigns against Chagas disease vectors have considerably reduced its prevalence in recent decades by eliminating populations of the most common vector species in human habitats. Vector transmission by *Triatoma infestans*, a non native vector introduced from Bolivia, has been officially interrupted in Brazil [2] and the same achievement has been obtained for *Rhodnius prolixus* in parts of the Andean Pact and Central America [3,4]. But, there were some endemic states in Brazilian Northeastern where *T*. *infestans* has never reached, as it is the case of Paraiba (PB), Ceara (CE) and Rio Grande do Norte (RN) [5]. In this region, autochthonous vectors are present, including *T*. *brasiliensis*, that is the predominant species found inside domiciles. His involvement in the hyperendemic transmission foci of Chagas disease in RN is pointed out since Lucena’s works in 1970 [6], that is before *T*. *infestans* elimination. However, since the strictly intradomiciliary *T*. *infestans* vector is eliminated, investment in vector control and surveillance has decreased in Brazil. Furthermore other triatomine specie—like *T*. *brasiliensis—*have been able to expand and colonize domiciliary and peridomiciliary environments from sylvatic ones [5,7–10]. We have a critical need to understand how vectors adapt to human environments—a process called domiciliation—to control the spread of Chagas disease. The domiciliation process is considered as a gradual process that may have been happening to many species, and it was taken as one of the main scientific challenges for the next decades related to vector surveillance [4].

The insect chemosensory system plays a key role in ecological adaptation to new or changing hosts or habitats [11]. At the molecular level, chemical recognition depends on the activation of specific sets of genes, including genes encoding odorant-binding proteins (OBPs), chemosensory proteins (CSPs), olfactory receptors (ORs) and gustatory receptors (GRs) (reviewed in [12]). OBPs and CSPs are soluble, secreted proteins that appear to play a role in ligand binding and transport to membrane receptors. ORs and GRs recognize specific ligands and transform the chemical signal into an electrical signal that will be transmitted to the brain, leading to the insect response [13–15]. Insect OBPs [16], ORs [17] and GRs [18] evolve rapidly via gene duplication/loss events, in parallel with adaptation to new ecological niches. Changes in olfactory sensitivity can be driven by these gain and loss events but also by intragenic mutations [19] or variation in gene expression [20].

The insect odorant landscape also includes pheromones—some crucial for mating [21], as demonstrated for *T*. *brasiliensis* [22]. The expression of transport proteins of these specific odorants may therefore differ between sexes [23–28]. When their environment changes, organisms respond by tuning gene expression. Rapid response to a brief, stressful event can persist as a long-term adaptation to a selective pressure [29]. A change in gene expression is a major component of genetic modulation in phenotypic evolution [30]. New generations of sequencing have considerably expanded opportunities to explore transcriptomes of non-model organisms using RNA-seq. This revolutionary tool provides unprecedented precision in the measurement of transcript levels [31].

The aim of the present study was to detect differentially expressed genes that play a role in the domiciliation process and sexual behavior of *T*. *brasiliensis* bugs sampled in different ecotopes (sylvatic, peridomiciliary, domiciliary). We first evaluated the diversity of OBPs and CSPs through the analyses of a *T*. *brasiliensis* reference transcriptome [32] and compared this diversity within the super-order Paraneoptera by building protein phylogenetic trees. We then evaluated contigs that were significantly differentially expressed (DE) in different environmental conditions and searched for contig clusters that show similar expression patterns using HTSCluster. We evidenced genes significantly differentially expressed between sexes and ecotopes including genes belonging to the chemosensory system (especially *OBP* and *CSP* genes), genes encoding takeout proteins involved in adult feeding and male courtship behavior, or genes encoding for proteins involved in detoxification or in preventing toxins from penetrating the cuticle.

## Materials and methods

### Sampling, RNA extraction and sequencing

*T*. *brasiliensis* individuals were collected in March 2011 in Caicó city, Rio Grande do Norte, Brazil (from 06 23 12.6 to 06 41 58.0 S and 37 04 47.3 to 37 12 08.0 W; Table 1), within the *Caatinga* ecoregion [33]: i) domiciliary bugs were sampled in various localities (B, J, P, R, T, U) in the indoor spaces of homes where triatomines are generally found in the crevices of mud walls, in furniture and under beds; ii) peridomiciliary bugs was sampled in the D locality in areas outside and within approximately 100 m of homes, where domesticated animals sleep or are maintained, namely in our study in henhouses; and iii) sylvatic bugs were sampled in sylvatic areas (A, C) in the Environmental Conserved Area (ECA) of Caicó that is under the supervision of military guards. The maximal linear distance is about 36 kms (between B and R, T, U), and the minimal linear distance is between A and C (about 1.5 km). Domiciliary and peridomiciliary samples were collected in the daytime; sylvatic samples were collected at night but all were sacrificed at the same time. We obtained permission from house owners/residents to collect insects from all homes and properties.

**Table 1:** *T*. *brasiliensis* sampling.

Geographic index	Locality	Environment	Geographic coord. (S/W)	Female NS/T(%NS)	Male NS/T(%NS)	Total pop NS/T(%NS)
B	São João do Sabugi	D	06 41 58.0 / 37 10 22.6		1/3 (33)	
J	Caicó/downtown	D	06 28 24.5 / 37 05 25.9	1/1(100)		
P	São Fernando	D	06 23 12.6 / 37 12 08.0	0/1 (0)		
R	Caicó/Sino	D	06 32 30.6 / 37 04 47.3		0/1 (0)	
T	São Fernando	D	06 23 16.1 / 37 12 04.1	1/1 (100)		
U	São Fernando	D	06 23 16.1 / 37 12 04.1	0/1 (0)	1/1 (100)	
B-U		D	06 23 12.6 to 06 41 58.0 / 37 04 47.3 to 37 12 08.0	2/4 (50)	2/5 (40)	4/9 (44.5)
D	Caicó/Sino	P	06 32 23.4 / 37 05 00.0	2/6 (33.5)	5/11 (45.5)	7/17 (41)
A	Caicó/EPA	S	06 28 25.0 / 37 05 21.4	2/8 (25)	2/10 (20)	4/18 (22)
C	Caicó/EPA	S	06 28 21.6 / 37 05 12.5	3/4 (75)	12/15 (80)	15/19 (80)

Geographic index, locality, environnement (with D = domiciliary, P = peridomiciliary, S = sylvatic) and geographic coordinates are provided; the number of not starved (NS) individuals / the total number of individuals is given per sex and per sample. The percentage of not starved individuals is in brackets.

For the domiciliary bugs, they were merged in a single sample named B-U. For all samples (B-U, D, A, C), only adults were used and separated according to sex. The heads were placed in RNAlater solution (Thermo Fisher Scientific) for RNA extractions. The body were placed in absolute ethanol for DNA extractions for population genetics studies and blood meal determination using molecular markers performed in [33]. For the all samples the nutritional status was determined as follows: if the molecular blood meal determination was not possible due to too little blood in its digestive tracts, the individual was considered as starved. For the sample A, 22% of the individuals were not starved, 44.5% for B-U, 80% for C and 41% for D. No major difference is notified between the nutritional status of males and females.

To target expressed chemosensory genes, we extracted RNA from the antennae and rostrum using the TRIzol Reagent kit (Invitrogen, Carlsbad, CA, USA). We pooled 4 to 15 individuals of the same sex and from the same sample to ensure there was enough RNA for sequencing and to measure average gene expression in a sample. We made technical replicates for samples with enough RNA (see details in Table 2). We sampled two biological replicates per sex for the sylvatic condition (SFA and SFC for females; SMA and SMC for males). Fourteen libraries were constructed using TruSeq RNA Kit 2010 from Illumina, and sequenced with the Illumina HiSeq 2000 method in single-reads of 100 bp on the LGC Genomics platform GmbH (Berlin, Germany).

**Table 2:** *T*. *brasiliensis* sample details and summary of RNAseq data.

Sample name	Environment	Sex	No. of individuals	No. of adapter-filtered reads			No. of mapped reads				
				Run1	Run2	Total	Run1	Run2	Total	Percentage reads mapped/total	Max counts (%)
DFB-U	D	F	4	9,210,196	13,482,702	22,692,898	1,391,136	1,985,127	3,376,263	14.88	28.41
DMB-U1	D	M	5	13,945,801	29,820,509	87,001,857	4,198,630	9,667,151	26,453,737	30.41	38.81
DMB-U2	D	M	5	24,053,273	19,182,274		6,852,782	5,735,174			
PFD1	P	F	6	7,024,781	15,993,588	45,152,589	2,728,046	6,627,049	17,319,989	38.36	13.51
PFD2	P	F	6	10,559,081	11,575,139		3,691,981	4,272,913			
PMD	P	M	11	1,851,110	4,771,764	6,622,874	550,846	1,460,386	2,011,232	30.37	34.49
SFA1	S	F	8	5,122,662	20,488,218	48,421,745	1,849,122	7,924,973	16,626,180	34.34	22.21
SFA2	S	F	8	7,782,766	15,028,099		2,229,032	4,623,053			
SMA1	S	M	10	25,231,786	3,319,544	74,845,155	9,460,295	1,285,816	27,848,905	37.21	28.87
SMA2	S	M	10	24,253,202	22,040,623		8,847,900	8,254,894			
SFC1	S	F	4	31,123,710	40,633,951	94,201,052	5,794,452	7,943,048	16,898,699	17.94	20.88
SFC2	S	F	4	3,421,756	19,021,635		494,700	2,666,499			
SMC1	S	M	15	13,953,748	21,062,918	46,892,268	4,876,329	8,285,962	19,181,873	40.91	46.26
SMC2	S	M	15	4,593,481	7,282,121		2,260,114	3,759,468			

For sample names we used: the environment (D = domiciliary, P = peridomiciliary or S = sylvatic); the sex (M = male or F = female); and the geographic index (B-U, D, A, C). The number at the end of the sample names indicate technical replicate number (no technical replicate was performed for DFB-U and PMD). RNA was extracted from antennae and rostra, sequenced in Illumina single-reads and mapped on the reference transcriptome. Number of filtered reads and number of mapped reads per sample are indicated. The Max_counts represents the percentage of reads mapped in the most expressed contigs.

The number of reads obtained for each sample, replicate and run is reported in the Table 2.

### De novo assembly of the reference transcriptome

We assembled the reference transcriptome from datasets consisting of one chemosensory library of a sylvatic female sequenced via Illumina paired-end and of eight chemosensory libraries from the males and females of the four samples described above pooled and sequenced with 454 technology. We followed the procedure 10c published in a previous study [32] with the following modifications: we cleaned paired-ends using prinseq version 0.20.4 [34] and we added the step of selecting the longest isoform per transcript cluster (as defined in Trinity) from the Trinity output. Transcriptome completeness was assessed using the CEGMA (Core Eukaryotic Genes Mapping Approach) pipeline that searches for the presence of sequences belonging to a set of ultra-conserved eukaryotic proteins [35].

### Annotation

We look at contigs that encode for a set of proteins of interest (CSPs, OBPs, ORs, ionotropic receptors (IRs), gustatory receptors (GRs), transient receptor potential channels (TRPs), sensory neuron membrane proteins (SNMPs), takeout proteins, cytochrome P450, gluthatione S-transferases, cuticular proteins) in the reference transcriptome. We queried insect amino acid sequences retrieved from GenBank against our reference transcriptome using Tblastn searches with an e-value threshold of 10^−6^. The selected contigs were aligned to the non-redundant protein database (Blastx with an e-value threshold of 10^−6^). Only contigs with at least one protein match from the families cited above were kept.

For the OBP and CSP protein families, contigs were translated and checked for the following: conservation of 6 (OBPs) and 4 (CSPs) cystein positions, the presence of α helices using PSIPRED [36], and the presence of a signal peptide using SignalP [37]. The OBP/CSP protein repertoires of *T*. *brasiliensis* were compared to those translated from genomes of several Paraneoptera: 27 OBPs and 19 CSPs from the triatome *R*. *prolixus* [12,38], 5 OBPs and 8 CSPs from the louse *Pediculus humanus* [39] and 19 OBPs and 13 CSPs from the pea aphid *Acyrthosiphon pisum* [40]. We added translated sequences from ESTs of two bugs from the Miridiae family: 13 OBP and 8 CSP sequences from *Adelphocoris lineolatus* [41–43] and 16 OBP and 12 CSP sequences from *Apolygus lucorum* [44–46]. Signal peptides were deleted before alignment using MAFFT version 7 [47] with the following options: E-INS-I with the BLOSUM62 scoring matrix and offset 0.1. We inspected the alignment by eye with BioEdit [48] and removed major gaps or sequences that were too short. We used ProtTest v3.2 [49] to predict the best-fit models for protein evolution. The LG model with estimated Gamma parameter was retained. The tree was built with PhyML v3.0 [50] with 100 bootstrap replicates and represented using iTOL web server [51].

### Mapping and counts

Much less RNA was extracted from the PMD sample than from others, resulting in a poor quality cDNA library. Consequently, this sample was excluded from the differential expression analysis and clustering study. Reads from other samples were mapped to the reference transcriptome after removing sequence adapters using BWA with default options [52]. Though we selected only one isoform per “gene” (the isoform states were assigned by Trinity), isoforms remained in our reference transcriptome. To avoid a resulting bias in our count analysis, we excluded reads with multiple hits. Count tables of reads were performed for each sample using SAMtools [53]. To evaluate the variation between technical replicates and sequencing runs, we proceeded to an ACP based on normalized counts with a rlog function of DESseq2. The ACP revealed high similarity between runs and technical replicates (S1 Fig) allowing them to be pooled in the subsequent analyses.

### Differential expression analysis

Two different statistical methods were used for differential expression analysis: DESeq2 package v. 1.6.3 [54] and EdgeR v. 3.8.5 [55]. For both packages, we filtered contigs with a low coverage using HTSfilter [56]. We used the DESeq normalization method and a threshold of five mapped reads on average. DESeq2 automatically evaluates normalization and dispersion but we turned off the independent filtering. We chose the RLE model for normalization and a robust estimation method to evaluate dispersion with EdgeR. In both cases, count data was modeled with a negative binomial distribution in a Generalized Linear Models (GLM) with two factors: environment and sex. For both methods, we controlled the factor “sex” when testing the factor “environment”. We compared the differential expression for the three modalities (sylvatic, peridomiciliary, domiciliary) of the “environment” factor and for the two modalities (male, female) of the “sex” factor. Our two-factor model (sex and environment) allowed R packages to assess changes that would be due to one or the other factor despite the absence of biological replicates per sex for domiciliary and peridomiciliary conditions. We applied the Benjamini-Hochberg correction [57] for multiple tests. The contigs were considered to be differentially expressed (DE) when the *Padj* was below 0.05. We selected contigs that were differentially expressed with both the EdgeR and DESeq2 methods. These contigs were annotated using blastx (BLAST 2.2.29+) with the non-redundant protein database (version May 2015) and we selected only the best matches that had an e-value below 10^−6^. The heatmap was built from chemosensory contig expression with DESeq2.

### Gene clustering based on transcriptomic data

Clusters of co-expressed contigs were searched using HTSCluster [58]. This R package implements a Poisson mixture model to cluster expression observations. Despite the use of TMM normalization, HTSCluster is sensitive to library size. The sample DFB-U was excluded from the analysis because of the lack of a technical replicate for this sample otherwise there would have been one quarter fewer reads mapped than other samples for which technical replicates were collapsed. The count table was first filtered to remove contigs with a normalized average below 5. We selected the slope heuristic DDSE method.

## Results

### Reference transcriptome

We selected only the biggest isoforms from Trinity “genes” using the assembly 10c workflow established by [32], and obtained a reference transcriptome of 48,290 contigs with an N50 of around 1,160 bp and a total length of 56,014,905 bp. The transcriptome appeared to be quite complete, with a CEGMA value of 94.35%. Males and females sampled from several samples were pooled to generate the 454 sequencing data so that the reference transcriptome represented all environmental conditions and sexes.

### Annotation in reference transcriptome

We obtained 25 and 16 contigs annotated as *CSPs* and *OBPs* (S1 Table) in the *T*. *brasiliensis* transcriptome. All CSP deduced proteins showed α helices, a signal peptide and four aligned cysteins (except for TbraCSP16). OBP proteins were less conserved. Nevertheless, the majority showed α helices, a signal peptide, and six aligned cysteins. All *OBP* and *CSP* contigs presented mapped reads in all samples (except for TbraOBP12 in the SMC sample), suggesting they are expressed in both sexes and under all environmental conditions.

**Fig 1:**
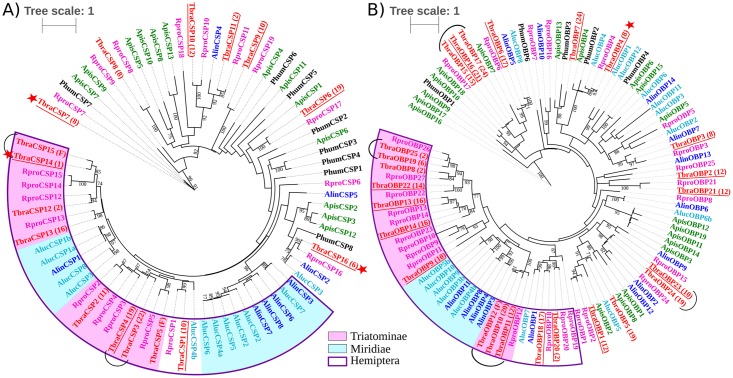
Phylogenetic tree of CSPs and OBPs in Paraneoptera. Maximum likelihood trees of a) CSPs and b) OBPs in Paraneoptera obtained either from genomic (G) or transcriptomic (T) data: *Rhodnius prolixus* (G, pink) *Triatoma brasiliensis* (T, red), *Adelphocoris lineolatus* (T, dark blue), *Apolygus lucorum* (T, light blue), *Pediculus humanus* (G, black) and *Acyrthosiphon pisum* (G, green). Bootstrap >70% are indicated. *T*. *brasiliensis* proteins whose contigs are differentially expressed between environmental conditions are underlined. *T*. *brasiliensis* proteins whose contigs are differentially expressed between sexes are indicated with a star. Braces represent clades of several *T*. *brasiliensis* proteins that are grouped with a single predicted *R*. *prolixus* protein, indicating polymorphism, alternative splicing of a single gene or paralogs. Boxes separate different clades and sub-clades (empty purple: Heteroptera, blue: Miridiae, pink: Triatominae). The information in parentheses refers to HTSCluster results. Numbers indicate to which cluster sequences belong. (F) indicates that the posterior probability (probability that a sequence belongs to a cluster) is less than 0.9.

Some *R*. *prolixus* proteins, have no tangible orthologs deduced from the *T*. *brasiliensis* chemosensory transcriptome (= RproCSP6, RproCSP9, RproCSP18, RproOBP5, RproOBP7, RproOBP8, RproOBP15, RproOBP16, RproOBP19, RproOBP25; Fig 1a and 1b). In the same way, no *R*. *prolixus* orthologs were found for two *T*. *brasiliensis* deduced proteins (= TbraOBP5 and TbraOBP7; Fig 1a and 1b). However, most of the OBPs and CSPs from *T*. *brasiliensis* have well supported orthologs deduced from the *R*. *prolixus* genome (for examples, several orthology relationships could be observed in CSP tree: RproCSP1 and TbraCSP1; RproCSP16 and TbraCSP16; RproCSP17 and TbraCSP6). Several OBPs and CSPs of *T*. *brasiliensis* are grouped with a single *R*. *prolixus* protein (for example, TbraOBP23 and TbraOBP24 were clustered with RproOBP24, see clades joined with a brace in Fig 1a and 1b). These proteins could be paralogs that arose from gene duplication specific to *T*. *brasiliensis*. In other cases, several transcripts of *T*. *brasiliensis* are grouped with several deduced proteins of *R*. *prolixus* (for example, TbraCSP3 and 4 were close to RproCSP3 and 4, two paralogs of *R*. *prolixus*). These proteins could be orthologs that arose from gene duplication in recent common ancestry of Triatominae. In such hypothesis (paralogy), each contig is supposed to represent a specific gene. However, as the *T*. *brasiliensis* chemosensory transcriptome was built from RNA pooled from several individuals, these proteins could also be encoded by allelic variants or isoforms from the same gene. Under these allelic or isoform hypothesis, all clustered contigs derive from the same gene. Therefore, the numbers of *OBP/CSP* genes expressed in antennae and rostra vary from 25 to 19 *OBPs* and 16 to 14 *CSPs*, depending on the hypothesis (paralogs *versus* alleles/isoforms). Hereafter, with the aim to limit overestimation of *OBP/CSP* gene numbers based on RNAseq database, we will only reference the lower and more stringent estimate of gene numbers (allelic/isoform hypothesis).

Because some *OBP/CSP* genes may not be expressed in selected tissues or stage used for samples, we can have underestimated the number of these gene in species from which only RNA data was available (*T*. *brasiliensis*, *A*. *lucorum* and *A*. *lineolutus*) compared to species from which genomic data was used (*R*. *prolixus*, *A*. *pisum* and *P*. *humanus*). Despite this underestimation, a higher number of OBPs and CSPs can be observed in the phylogeny of bloodsucking bugs (see pink blocks, Fig 1a and 1b) and more generally in Hemiptera (purple empty block, Fig 1a and 1b) compared to the other species of Paraneoptera. Enlarged genomic sample of Paraneoptera species would be required to validate this preliminary observation to infer genome evolution scenarios. Only 3 contigs encoding for odorant receptors (ORs), could be annotated comprising one Co-odorant receptor (Co-OR; S2 Table). Except Co-OR, ORs are poorly expressed compared to other chemosensory genes. Consequently, too few reads are sequenced from ORs transcripts to be *de novo* assembled explaining the low number of contigs annotated as ORs. Probably for the same reason, we didn’t found any ionotropic receptors (IRs) and gustatory receptors (GRs). We also annotated contigs that encode for the following proteins: transient receptor potential channels (TRPs) indispensable for the perception of the environment (n = 5); sensory neuron membrane proteins (SNMPs) potentially involved in detection of pheromones (n = 1); takeout proteins involved in the integration of processes related to circadian rhythm, in feeding-relevant activities and in male courtship behavior (n = 25); proteins involved in protection against toxin penetration or in detoxification-like cuticle proteins (n = 49); cytochrome P450s (n = 72); and glutathione S-transferases (n = 5) (S2 Table).

### Sequencing, mapping and counts

The sequencing depth varied a lot between samples: from 22,692,898 (DFB-U) to 94,201,052 reads (SFC), excluding the cDNA library of the PMD sample (Table 2). The library size (total number of mapped reads) which depends both on sequencing depth and on the percentage of mapped reads, also varied between samples. For example, only 14.88% of DFB-U reads were mapped compared to 40.91% of SMC. Overall, the library size included between 3,376,263 and 27,848,905 mapped reads.

Most of the mapped reads were captured by very few genes, which is quite common in RNAseq analysis. The maximum percent of reads mapped to a single contig varied from 13.51% (PFD) to 46.26% (SMC) (Table 2). The most expressed contig was the same in all samples except DFB-U. It was associated by blast with a putative uncharacterized protein (gi:133916482) of the immune-related transcriptome of *Thermobia domestica* [59].

### Differential expression analysis

**Fig 2:**
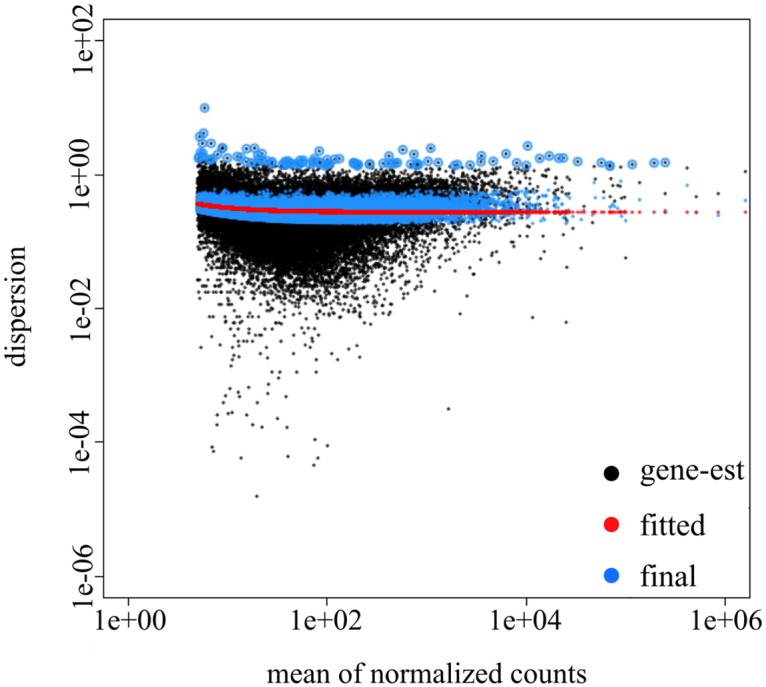
Dispersion estimates from DESeq2 and gene count measures between samples. Dispersion of each gene (black), the trend line for all samples (red), the corrected value of the dispersion (blue) and outliers (black dot surrounded in blue) are shown. Variance decreases with the number of reads per contig until it stabilizes.

**Fig 3:**
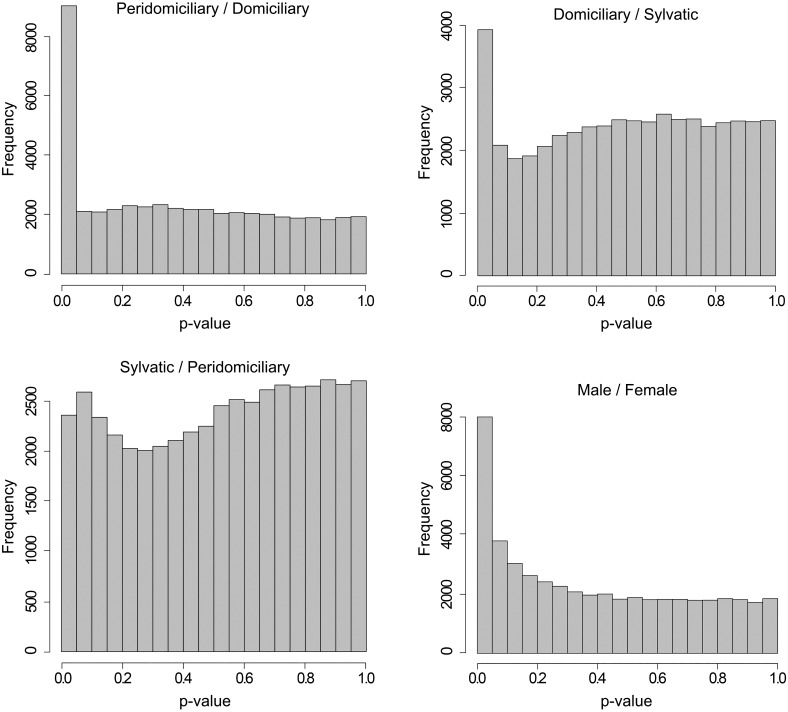
*P*-value distribution of differential expression analysis (DESeq2) per comparison

We used both DESeq2 and EdgeR software, which differ in dispersion estimation methods (see Fig 2 for DESeq2). For both software, dispersion decreases when expression increases, which is typical for RNAseq data among biological replicates. We used a two-factor general linear model (GLM) with both packages, allowing differential analysis despite the lack of biological replicates for some conditions. DESeq2 and EdgeR presented similar *p-value* distributions for all but the comparison between the sylvatic and peridomiciliary habitats. The peak was close to 0, corresponding to differentially expressed contigs between modalities and a uniform distribution for larger *p*-values, indicating a good fit to models generated by both types of software (Fig 3). However, the number of DE contigs differed between the two types of software. To increase the reliability of our results, we selected contigs in which we found DE with both EdgeR and DESeq2 (Table 3). We found numerous DE contigs (n = 148) between males and females (Table 3). More DE contigs (n = 3875) were found between peridomiciliary and domiciliary samples than between sylvatic and domiciliary samples (n = 29) and between sylvatic and peridomiciliary samples (n = 29). Among DE contigs, most were over-expressed in the chemosensory organs of the peridomiciliary bugs, while they were mainly under-expressed in those of the domiciliary bugs.

**Table 3:** Differential expression analysis.

Condition comparisons	DESeq2			EdgeR			Found in both DESeq2 and EdgeR			
	+	-	total	+	-	total	+	-	total	genes of interest
M/F	148	4	152	1537	636	2173	144	4	148	2 *CSPs*; 1 *OBPs*; 3 *TOs*; 3 *P450s*; 14 *CPs*
S/P	5	26	31	20	383	403	3	26	29	2 *CPs*
D/S	11	292	303	2	30	32	2	27	29	3 *OBPs*; 4 *P450s*
D/P	19	4062	4081	427	5756	7183	16	3859	3875	13 *CSPs*; 17 *OBPs*; 14 *TOs*; 3 *TRPs;* 1 *SNMP;* 16 *CPs*; 3 *GSTs;* 37 *P450s*

Results are indicated for DESeq2 and EdgeR. We selected contigs that both packages identified as differentially expressed. “+” indicates number of over-expressed contigs in the first modality listed in each condition comparison (example, 148 contigs are found as over-expressed in males compared with females using DESeq2). “-” indicates the number of under-expressed contigs. We annotated differentially expressed contigs found in both DESeq2 and EdgeR and we indicated the number of transcripts encoding odorant-binding proteins (OBPs), chemosensory proteins (CSPs), takeout proteins (TOs), transient receptor potential channels (TRPs), sensory neuron membrane protein (SNMP), cuticular proteins (CPs), gluthatione S-transferases (GSTs) and Cytochrome P450 (P450s). D = domiciliary, P = peridomiciliary and S = sylvatic; M = male and F = female.

### Annotation of differentially expressed genes

**Fig 4:**
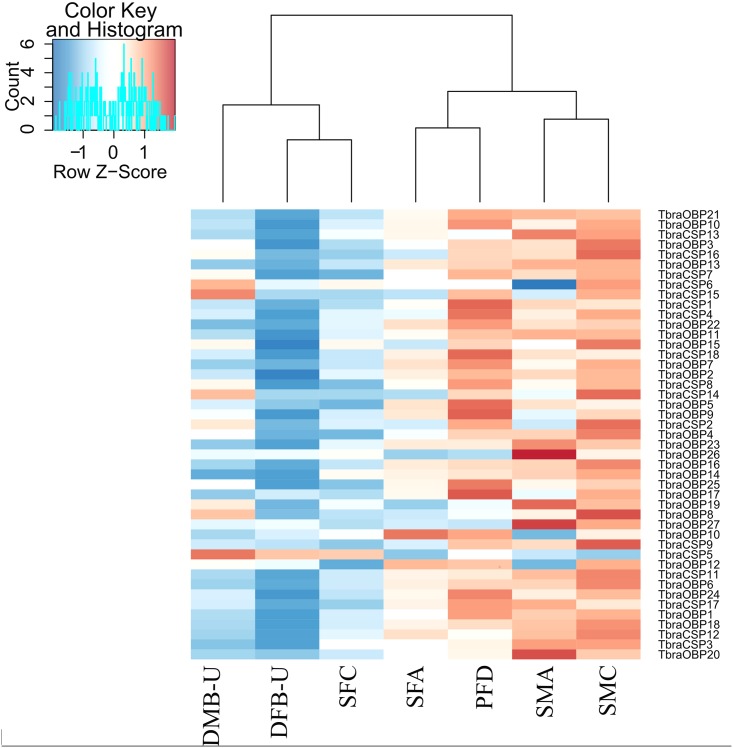
Heatmaps showing the sample distance of the expression data of differentially expressed *OBP/CSP* contigs (DESeq2).

We were only able to annotate 50% of the DE contigs with the non-redundant proteins database. Three *CSPs* (*CSP14*, *15* and *16*) and one *OBP* (*OBP4*) were differentially expressed between sexes, and all were over-expressed in males. A significant proportion of *OBP* and *CSP* contigs were differentially expressed between environments, particularly in the peridomiciliary-domiciliary comparison (S2 Table). Most were under-expressed in the domiciliary samples. However, no expression difference was observed for contigs annotated as ORs. We used a heatmap to compare chemosensory contig expression (Fig 4), revealing two distinct groups: one merged sylvatic and peridomiciliary samples and the other merged domiciliary samples plus the sylvatic SFC sample. The similarity between gene expression in domiciliary samples and this peculiar sylvatic sample could explain why few DE contigs were detected in the sylvatic *versus* domiciliary comparison.

Among differentially expressed contigs, we also found contigs that encode TRPs (n = 3), SNMP (n = 1), takeout proteins (n = 16), cytochrome P450s (n = 38), glutathione S-transferases (n = 3) and cuticle proteins (n = 22) (S2 Table). These genes were under-expressed in females compared to males and in domiciliary samples compared to peridomiciliary and sylvatic ones. However, some cuticle proteins were over-expressed in peridomiciliary samples compared to sylvatic ones.

### Gene Clustering

**Fig 5:**
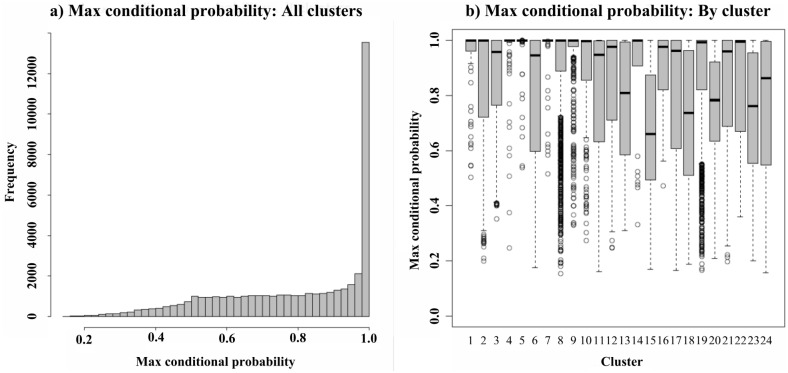
Histogram (a) of maximum conditional probabilities of cluster membership for all genes and (b) Boxplots of maximum conditional probabilities that genes assigned to each cluster (HTSCluster) are actually a member of that cluster.

**Fig 6:**
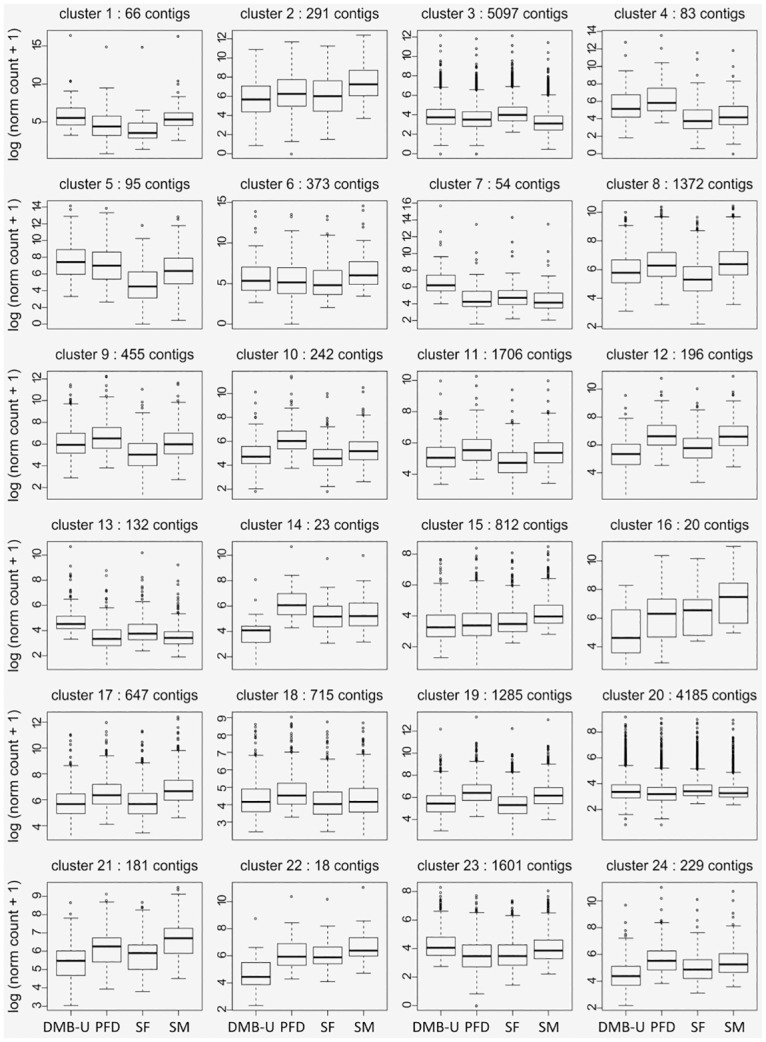
Distribution of contig expression in *T*. *brasiliensis* according to sex and environment for the 24 clusters defined by HTSCluster. Boxplot of the logarithm of normalized count data is shown. S = sylvatic, P = peridomiciliary and D = domiciliary. M = male and F = female. Contigs included in this analysis are those with a posterior probability higher than 0.9.

We selected the slope heuristic DDSE methods from HTSCluster to estimate the number of clusters because BIC and ICL did not converge. We detected 24 clusters containing 28 to 14,385 contigs per cluster. Most contigs were reliably classified in a cluster with a maximum conditional probability close to 1 (Fig 5a). The quality of this classification, however, varied between clusters (Fig 5b). For example, only 21.65% of the contigs were classified in cluster 15 with a maximum conditional probability greater than 0.9, while 91.35% of contigs were classified in cluster 5 with the same probability. Clustering results agreed globally with results obtained from the differential expression study. For example, clusters 12 and 14 were made up primarily of contigs that were under-expressed in the domiciliary environment (Fig 6). Most contigs belonging to clusters 12 and 14 were significantly under-expressed in domiciliary/sylvatic or domiciliary/peridomiciliary comparisons with both DESeq2 and EdgeR.

Interestingly, clustering results may also provide more subtleties. For example, DE analysis revealed very few contigs that were over-expressed in females and in domiciliary samples. Nevertheless, clustering analysis revealed that contigs in clusters 3 and 20 showed higher expression in sylvatic females compared to all other samples (Fig 3). Similarly, contigs in clusters 1, 5, 7, 13 and 23 showed higher expression in the domiciliary sample than in the sylvatic/peridomiciliary samples (Fig 3). This apparent discrepancy may have arisen because over expression was too slight to be detected in the DE study. Joint analysis of several contigs with similar expression patterns would help to detect if this were the case. However, contigs with similar patterns of DE between modalities may be split into several clusters based on estimates of their expression pattern distribution. For example, this was the case for *TbraOBP23* and *TbraOBP24*, which have very similar sequences (grouped with *Rhodnius prolixus* OBP24 in the phylogenetic tree) and are both over-expressed in the peridomiciliary sample compared to the domiciliary sample. They were, however, allocated to clusters 10 and 19, respectively. Similar conclusions can be drawn for *TbraCSP3* and *TbraCSP4*.

Unfortunately, we were not able to test gene ontology enrichment of the cluster because of the low proportion of the transcriptome annotated (*T*. *brasiliensis* is quite distant from *R*. *prolixus* for which a reference genome is available).

## Discussion

In Brazil, *T*. *brasiliensis* is the main concern for re-emerging hyperendemic foci of Chagas disease in semi-arid zones because this vector is found in a variety of ecotopes, namely g sylvatic, peridomestic and domestic ones. In this last environment *T*. *brasiliensis* is included in a restrict list of Brazilian native domiciliated species, along with *P*. *megistus*, *T*. *pseudomaculata* and *T*. *sordida* [5]. Odor stimuli are important in Chagas disease vectors and because there are remarkable distinctions between ecotopes it is expected that their capacity to adapt to a new habitat is linked to changes in chemosensory system genes.

### Reference transcriptome, CSP/OBP annotation

We estimated at least 19 *OBP* and 14 *CSP* genes from the *T*. *brasiliensis* reference transcriptome. Compared to annotated genes in the *R*. *prolixus* genome (27 *OBPs* and 19 *CSPs*) [38], about 85% of the potential gene repertoire was retrieved from *T*. *brasiliensis* chemosensory transcriptome. Annotation of *T*. *brasilienis OBP/CSP* genes might be incomplete because the transcriptome was performed from antennae and rostrum while some *OBPs* and *CSPs* could be specifically expressed in other tissues. Therefore the total number of *CSP* and *OBP* genes occurring in the *T*. *brasiliensis* genome is likely higher than reported here. Interestingly, two *T*. *brasiliensis* OBPs have no orthologs in the *R*. *prolixus* genome. As they are clustered with OBPs from other species (*A*. *lucorum* and *P*. *humanus*), these copies could have been lost secondarily in *R*. *prolixus*.

Our results confirm the hypothesis that bloodsucking bugs have more *OBP* and *CSP* genes than other Paraneoptera, especially *A*. *pisum* (19 *OBPs* and 13 *CSPs*) and *P*. *humanus* (5 *OBPs* and 8 *CSPs*). How specialized a species is could be linked to how many *OBP* and *CSP* genes it has. The insect chemosensory genes are known to have evolved rapidly via gene duplication or loss events, in parallel with adaptation to new ecological niches [15–17,60,61]. The stable environment that results from a host-dependent lifestyle could explain why *A*. *pisum* and *P*. *humanus* have fewer OBP and CSP genes [39,40]. Two hypotheses might explain the OBP and CSP expansions in Chagas disease vectors that may be related to i) recruitment for a derived function, and more specifically in relation to hematophagy or ii) new chemosensory functions selected in response to environmental change. Striking similarities between OBPs and heme-binding proteins—like the occurrence of a signal peptide, six cysteins and a conserved PBP_GOBP domain [12]—support the hypothesis that *OBP* and *CSP* gene expansion is an adaptation to hematophagia. Some OBPs could have a derived function in providing protection from oxidative stress by binding the heme from digested blood hemoglobin, like heme-binding proteins [62]. Moreover, some CSPs share an OS-D domain—a chemical characteristic of lipocalins—with proteins encoded by genes found in the salivary transcriptome of another bloodsucking bug, *Triatoma matogrossensis*. Lipocalins are a class of proteins secreted in the saliva of bloodsucking bugs. They serve primarily as carriers of small ligands, and are used to disarm the host hemostatic machinery [63]. Some CSPs may play a similar role [12]. Further studies are needed to explore such derived functions of olfactory/chemosensory proteins in bloodsucking bugs.

In Chagas disease vectors, gene expansion could also reflect a process of selecting new chemosensory functions, providing an evolutionary advantage to individuals able to recognize various odor stimuli. Indeed, Triatominae are associated with diverse habitats, namely vertebrate nests or burrows in sylvatic habitats found in trees, palms, bromeliads or rocks, cracks and crevices in anthropogenic habitats such as corrals, chicken coops or human dwellings. The Triatominae feed on a broad range of vertebrate hosts; 1150 vertebrate host species have been described [64]. For feeding sources detected for *T*. *brasiliensis* in Caico [33], a species of sylvatic rodent (*Kerodon rupestris*) was prevalent in conserved sylvatic area whereas synantropic (*Galea spixii*) or domestic animals (*Capra hircus* and *Gallus gallus*) were prevalent for anthropogenic systems (peridomiciliary and domiciliary environments). Thus, the diversity of OBPs and CSPs could be explained as adaptation in blood-sucking insects that live in a putative diversity of ecological niches.

### Differential gene expression

To maximize support for our results, we selected contigs that the EdgeR and DESeq2 techniques both identified as differentially expressed. Recent studies suggest that EdgeR and DESeq2 R packages are the best statistical methods for normalization and differential expression analysis of multi-factored experiments without a reference genome [58,59,65,66]. They are currently the only methods able to maintain a reasonable false-positive rate without decreasing the power of differentially expressed gene detection. They are stable under different sequencing depths. However, DESeq2 and EdgeR did not provide the same results with our dataset. EdegR found more differentially expressed contigs than did DESeq2. Moreover, EdgeR found a higher log2-fold change than did DESeq2. Nevertheless, RNAseq is a recent technology and statistical research into how best to use differential expression analysis software with this kind of data is ongoing.

Recently, a gene clustering methodology based on transcriptomic data has been developed to highlight co-expressed contigs (HTSCluster, [58]). We found similar results using differential expression analysis and the HTSCluster gene clustering methodology. Furthermore, gene clustering provides additional detail about the dynamics of gene expression, especially for chemosensory contigs. We were able to annotate *OBP* or *CSP* contigs with very similar sequences to different clusters that reflect different expression patterns, suggesting they represent recent paralogs or isoforms rather than allelic variants. This could support the idea that these sequences play a role in different functions, for instance they could target different odorant molecules.

### Differential expression between sexes

We found 148 contigs to be differentially expressed between males and females. These were probably genes involved in sex-specific behavior or sexual dimorphism.

One *OBP* and three *CSPs* were all over-expressed in males. Authors proposed that only male *T*. *brasiliensis* are attracted by sexual signals [22]. *OBPs* and *CSPs* that are over-expressed in males in our study could be involved in this kind of sexually dimorphic behavior. Previous studies of *A*. *lucorum* and *A*. *lineolatus*, two species that were included in the phylogenetic tree, showed that most of the *OBPs* and *CSPs* are over-expressed in female *A*. *lucorum* antennae. [45,46] while *OBP* or *CSP* over-expression may occur in either males or females in *A*. *lineolatus* [41–43]. This suggests that expression and function of these proteins vary within Hemiptera.

Three contigs annotated as *takeout* were also over-expressed in males. *Takeout* genes have been shown to affect male courtship [67] and sexual dimorphism in locomotor activity, modulating the circulating juvenile hormone level in *Drosophila melanogaster* [68]. Locomotor activity also varies between sexes in *T*. *infestans*, where females disperse more than males both by walking [69] and flying [70]. In contrast, dispersal activity seems to be higher in male than in female *T*. *brasiliensis*: more males than females could be collected when using a flashlight to capture dispersing individuals [71]. Thus, differential courtship or sexual locomotor activity between sexes could explain the over-expression of *takeout* genes observed in *T*. *brasiliensis*.

We also detected differences between sexes in expression of contigs involved in insecticide resistance, e.g. genes encoding P450s and cuticle proteins. The *cytochrome P450* gene family is implicated in several functions other than detoxification. For example, in *D*. *melanogaster*, a *cytochrome P450 (cyp4d21)* named *sxe1* is differentially expressed between males and females and identified as a circadian-regulated gene involved in male courtship and mating success [72]. Differences in the ratio of cuticular proteins have also been found in the thoracic cuticle of male and female crickets (*Schistocerca gregaria* and *Locusta migratoria*). This difference has been linked to the specific mechanical ability of females to stretch intersegmental membranes [73]. Likewise, six genes that encode cuticular proteins were differentially expressed between the two sexes of *Anopheles gambiae*, which is probably linked to the sex-specific hematophagous behavior in this species [74].

### Differential expression between environments: under-expression of contigs in domiciliary bugs

Our study revealed numerous differentially expressed genes in the transcriptomes of bugs sampled in different environments. This could either be explained by genetic adaptation to an anthropogenic system or by a phenotypic plasticity due to changes in gene expression induced by local environmental conditions. Whatever the mechanism, as the peridomiciliary environment is human-modified, and because it is commonly assumed that the direction of re-colonization of homes after insecticide spraying is likely to be from peridomiciliary to domiciliary environments [75], we expected more similar phenotypes in the domiciliary and peridomiciliary samples (e.g. under- or over-expression of the same set of genes). But, our comparison of peridomiciliary against domiciliary samples revealed much more differentially expressed contigs than did a comparison of sylvatic against domiciliary or peridomiciliary samples. However, our results are congruent with the population genetics and eco-epidemiologic data previously obtained on *T*.*brasiliensis* populations evidencing that i) the sylvatic and domiciliary cycles are genetically connected, ii) the co-occurrence of two *T*.*cruzi* strains in sylvatic *T*. *brasiliensis* population that is also consistent with a link between sylvatic and domiciliary cycles, and iii) the preponderance of *G*. *spixii* in the *T*. *brasiliensis* feeding source in peridomiciliary areas revealing that this rodent is no longer only sylvatic but become a highly synantropic animal [33].

Although domiciliary individuals were sampled in different localities, which could have resulted in expression variability, a difference in gene expression is nevertheless observed, most DE genes were under-expressed in this environment compared to the others, probably reflecting a less variable and more predictable biotope (abiotic and biotic conditions). A single sylvatic sample is probably responsible for the low number of DE genes detected between sylvatic and domiciliary samples in our study: patterns of expression in the sample SFC (females from sylvatic sample C) were clearly more similar to those of domiciliary samples than to other sylvatic ones.

### Expression of chemosensory genes

Contigs annotated as *OBPs* and *CSPs* followed the general trend of under-expression in the domiciliary samples. Poor biotic diversity probably led to lower diversity of odorant stimuli in the domiciliary environment than in others. This reduction of stimuli could explain the under-expression of chemosensory genes in domiciliary bugs. Furthermore, several studies have linked chemosensory gene expression to feeding status. In the fly *Glossina morsitans*, transcription of CSP genes was related to host searching behavior [76]. A recent study of bloodsucking bugs [77] revealed that, in *R*. *prolixus*, some odorant and ionotropic coreceptor genes are less expressed after a blood feeding. Similar results were previously found in *An*. *gambiae* [78]. Concerning the *T*. *brasiliensis* samples, their nutritional status was somewhat similar except the sylvatic sample (C) that was the less starved. Thus, the nutritional status of the bugs when they were sacrificed is not the most likely factor to explain the differential expression of the OBP/CPS genes in the domiciliary sample, but it cannot be entirely discarded.

### Expression of *takeout* gene families

*Takeout* genes are also expressed in chemosensory organs and have been proposed to be associated with chemosensory perception in both taste and olfactory systems [79]. Takeout proteins link temporal cycle and feeding status information that are involved in the circadian cycle. They are involved in feeding-related metabolism and behavior [67,80] and in the regulation of locomotor activity during foraging [68]. Seven contigs were described as belonging to the *takeout* gene family in a transcriptome study of the digestive tract of *R*. *prolixus* [81], but until now no *takeout* genes had been annotated in *Triatoma*. We found a high number of *takeout* genes in *T*. *brasiliensis* that were differentially expressed according to the environments. The nocturnal activity pattern displayed by adult triatomine bugs is described as generally bimodal with the first peak just after dusk associated with host-seeking activities and the second at dawn, with the search of an appropriate daytime shelter [82–84]. Some authors correlate host searching induced by the nutritional status of the bug with the dispersion process that may lead Chagas disease vectors to colonize domiciliary habitats [71,85,86]. Because the nutritional status of the domiciliary sample is similar to that of two others samples (peridomiciliar and sylvatic A), a lower foraging activity is again more likely, linked to the higher host availability in domiciliary habitats.

### Expression of genes encoding Sensory Neuron Membrane Proteins and Transient Receptor Potential channels

The sensory neuron membrane proteins (SNMPs) were recently suggested to play a significant role in insect chemoreception. Insect SNMPs are two transmembrane domain-containing proteins in olfactory neurons of antennae and localized in dendrite membranes. They were first identified in the pheromone-sensitive hairs of the wild silk moth *Antheraea polyphemus* [87] and tobacco hornmoth *Manduca sexta* [88,89], and now are described in various insect orders namely including Lepidoptera, Coleoptera, Hymenoptera and Diptera [90] and recently Orthoptera [91]. Several functions of insect SNMPs in odorant detection have been predicted (reviewed in [90]) including interactions with pheromone binding protein (PBP)-pheromone complexes, OBP/odorant complexes, OR proteins. Moreover, it was demonstrated in drosophila, that SNMPs regulates sexual and social aggregation behaviours [92,93]. Transient Receptor Potential (TRP) superfamily of ion channels is essential for the perception of environment. In insects, TRPs are involved in phototransduction, thermosensation, vision, hygrosensation, mechanosensation and also in chemosensation, including taste and olfaction [94,95]. By their function, TRPs have an important impact on insect behaviour [96]. A recent study suggested that a TRP belonging to vanilloid receptors family (TRPVs) could be involved in the high sensitivity to heat in *R*. *prolixus* which uses these capacities to perceive warm-blooded hosts [97]. In *T*. *brasiliensis*, the unique SNMP annotated and three out the five TRP contigs annotated were over-expressed in peridomiciliary samples. By way of explanation, in henhouses, we can mention that, in *T*. *brasiliensis* and more strikingly in *T*. *pseudomaculata*, very strong triatomine aggregation is observed in their shelters (C. Almeida personal communication). One can assume that henhouses where host density is high compared to other habitats there is a higher sensory interference, so bugs produce aggregation pheromones in higher quantity in their shelters to better find them after their blood meal. This could results in *SNMP* over-expression in the peridomiciliary sample. Moreover, the average body temperature of chickens is 40–42°C, compared to the 37°C of mammal vertebrates. This higher temperature of chicken hosts could explain the *TRP* over expression in relation to a higher exposition to heat.

### Expression of genes involved in insecticide resistance

Regular insecticide spraying campaigns in Brazil have been used against triatomine bugs after the 1970s [98]. Although, all *T*. *brasiliensis* tested until now have been highly susceptible to deltamethrin, an alpha-cyano-substituted pyrethroid insecticide [99], insecticide resistance has been demonstrated for some Chagas disease vectors, including *T*. *infestans* [100–102]. We found DE genes involved in the insecticide resistance process, including genes encoding cytochrome P450s, gluthatione S-transferases and cuticle proteins. Accordingly, several studies of cytochrome P450s have revealed over-expression in field populations of insects that are resistant to insecticide [103–108]. Cytochrome P450 genes are involved in detoxification. They catalyze various reactions, e.g. mono-oxygenation [106,107]. The role of gluthatione S-transferases in detoxification is less understood. Several studies showed that they are linked to insecticide resistance and are over-expressed in pyrethroid-resistant insect populations [106,109–111].

Insecticide resistance could also come from an increased ability to prevent toxins from penetrating the cuticle. Thus, changes in cuticle conformation or thickness could increase insecticide resistance [106,112,113]. For example, over-transcription in a resistant strain of *An*. *gambiae* has suggested that the cuticle plays a role in response to selection pressures resulting from insecticide treatments [114]. In our study, cytochrome P450s, gluthatione S-transferases and cuticular proteins were all under-expressed in domiciliary bugs, which should be the most exposed to insecticide treatment. However, the domiciliary individuals were sampled in houses that had not been sprayed with insecticide for three years. Over-expression of these same genes in sylvatic and peridomiciliary bugs could reflect the involvement of detoxification enzymes in a large range of xenobiotics, including some that may differ according to the habitat [106]. In addition to their role in insecticide resistance, cuticle proteins also play a role in the response to environmental stresses [115]. The domiciliary environment probably has lower xenobiotic diversity and more stable abiotic parameters than do sylvatic and peridomiciliary environments. This could explain why both detoxification and cuticle protein genes were less expressed in domiciliary bugs.

## Conclusion

Our aim was to better understand the domiciliation process in *T*. *brasiliensis*. We focused on the chemosensory transcriptome because chemosensory genes are implicated in adaptation to changing environments. In the absence of a reference genome for *T*. *brasiliensis*, we used the methods of Marchant *et al*. [32] to build a reference transcriptome. RNAseq is a recent technology, so differential expression analysis software is still not optimized. Therefore, to generate results with strong support, we selected congruent results from two different programs: EdgeR and DESeq2. We then processed an independent clustering analysis based on the same expression pattern data. We characterized numerous DE transcripts from bugs of different environments and of different sexes. The domiciliary sample showed different expression profiles than those of sylvatic and peridomiciliary samples. Since few samples from domiciliary and peridomiciliary samples were analyzed, it is difficult to generalize our conclusions, but this study has identified new questions that need to be answered to understand the domiciliation process. Focusing on specific gene families, we highlighted the link between the environment from which a bug originates and expression of chemosensory genes (*OBPs*, *CSPs*), detoxification, cuticle protein and *takeout* genes. These are good candidate genes for playing a role in adaptation or in plastic rearrangement in response to environmental changes, which could include anthropic pressure. To generalize our results, we plan to develop specific primers for these candidate genes to evaluate their expression by qPCR on a wide range of domiciliary, peridomiciliary and sylvatic samples of *T*. *brasiliensis*. Further studies are also needed to understand the function of genes highlighted by our analysis and to propose scenarios of molecular evolution of multigenic gene families (paralogs, isoforms, allelic variants) that will require new data, especially genomics. Understanding the molecular basis of vector adaptation to human dwellings creates the potential to develop new tools for disease vector control, such as disrupting chemical communication.

## Supporting Information

S1 Fig ACP based on normalized counts with the rlog function of DESseq2. (TIF)

S2 Fig Diagnostic plots provided by the *capushe* package for the DDSE approach of HTSCluster. (TIF)

S1 Table Blast annotation of CSPs and OBPs, and information about differential expression status. Results of Blast: For all contigs that matched with a CSP or an OBP from the non-redundant protein database, we indicate the ID of this protein (Blast NR ID) and its denomination (Annotation). For each match, we provide the percentage of identity (%ident), the score and the e-value. Each contig that matches with an OBP or CSP was translated and the corresponding protein analyzed by checking for the number of conserved cysteins, the presence of peptide signal and the number of α-helices. For each contig annotated as OBP or CSP, we indicated in which comparison both DESeq2 and EdgeR found it to be differentially expressed. D = domiciliary, P = peridomiciliary and S = sylvatic; M = male and F = female. The log2fold and the adjusted *p*-value for both packages are provided, as well as the cluster number allocated by HTSCluster.
(DOCX)

S2 Table Blast annotation of ORs, TRPs, SNMPs, takeout, cytochrome P450, glutathione S-transferase and cuticular proteins and information about differential expression status. Results of Blast: For all contigs that matched with a protein of interest in the non-redundant protein database, we indicate the protein ID (Blast NR ID) and its denomination (Annotation). For each match, we provide the percentage of identity (%ident), the score and the e-value. For each contig, we indicate in which comparison both DESeq2 and EdgeR found it to be differentially expressed. D = domiciliary, P = peridomiciliary and S = sylvatic; M = male and F = female. The log2fold and the adjusted *p*-value for both packages are provided, as well as the cluster number allocated by HTSCluster.
(DOCX)
