# Supplementary figures and images for: Under-Expression of Chemosensory Genes in Domiciliary Bugs of the Chagas Disease Vector Triatoma brasiliensis
Source: PLoS Negl Trop Dis. 2016 Oct 28;10(10):e0005067. doi: 10.1371/journal.pntd.0005067 (PMC5085048; doi:10.1371/journal.pntd.0005067)

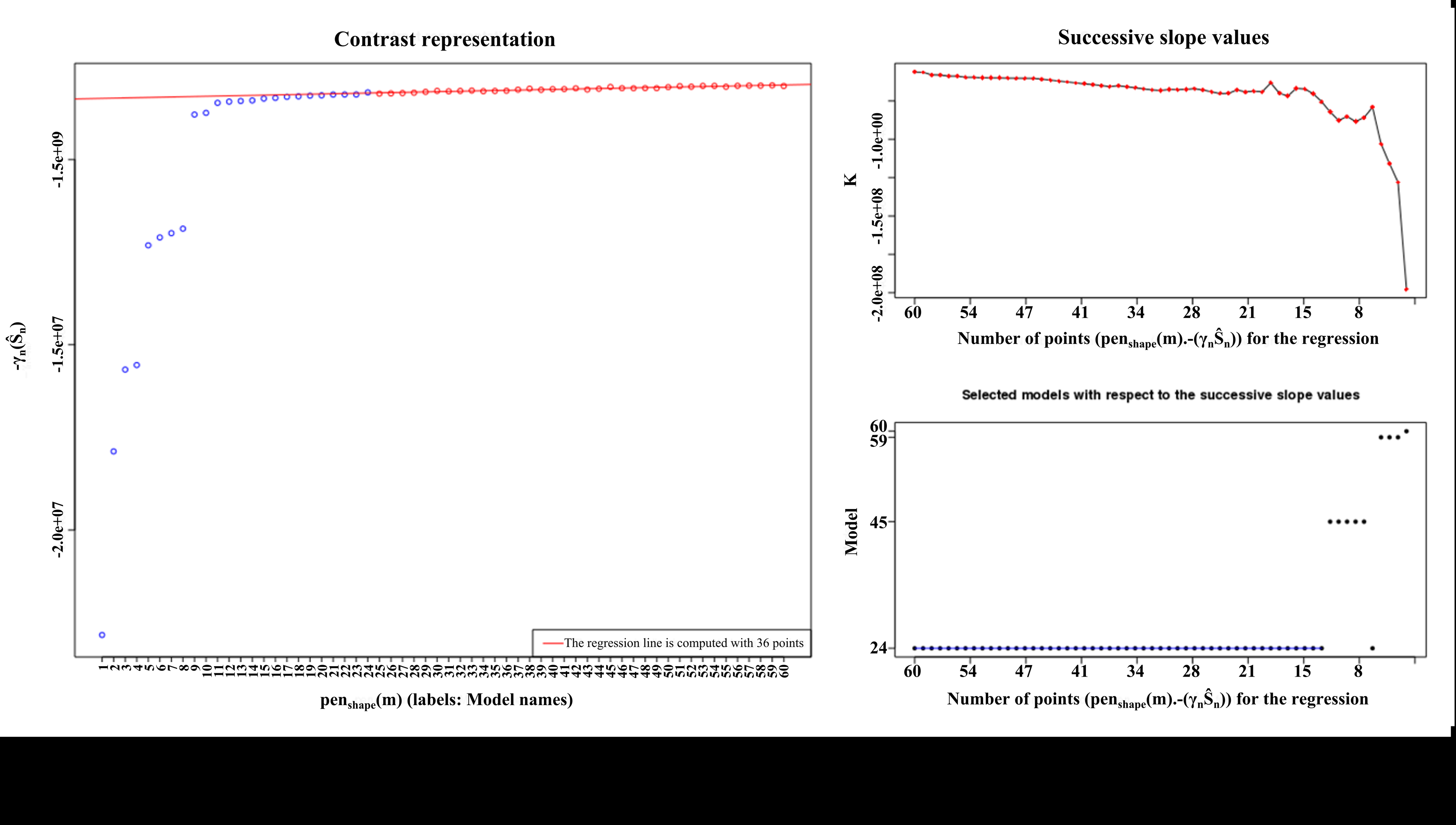

Supplement: S1 Fig — (TIF) [file pntd.0005067.s001.tif]

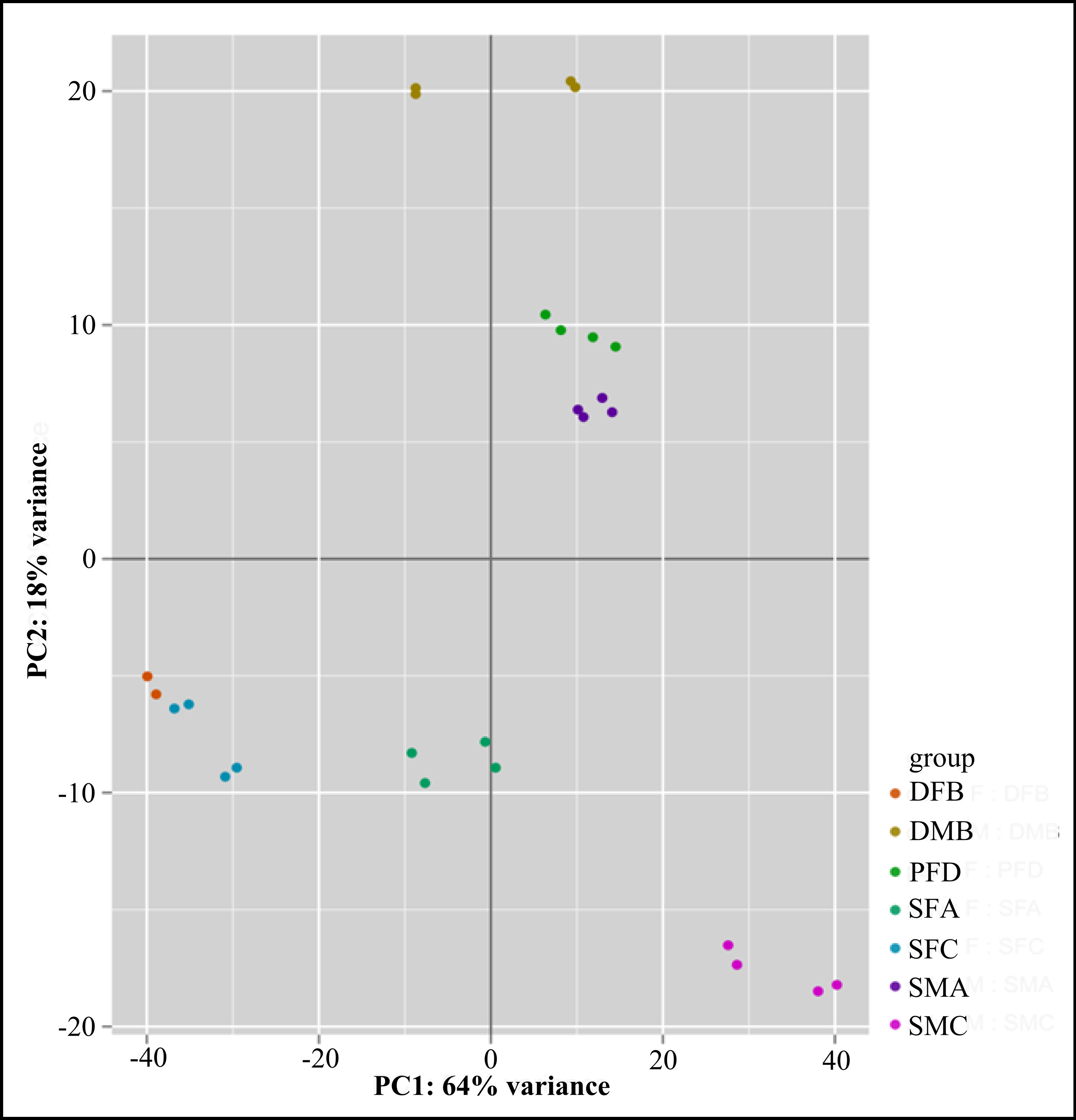

Supplement: S2 Fig — (TIF) [file pntd.0005067.s002.tif]
