# Supplementary material for: Under-Expression of Chemosensory Genes in Domiciliary Bugs of the Chagas Disease Vector Triatoma brasiliensis
Source: PLoS Negl Trop Dis. 2016 Oct 28;10(10):e0005067. doi: 10.1371/journal.pntd.0005067 (PMC5085048; doi:10.1371/journal.pntd.0005067)
Supplement: S1 Table — Results of Blast: For all contigs that matched with a CSP or an OBP from the non-redundant protein database, we indicate the ID of this protein (Blast NR ID) and its denomination (Annotation). For each match, we provide the percentage of identity (%ident), the score and the e-value. Each contig that matches with an OBP or CSP was translated and the corresponding protein analyzed by checking for the number of conserved cysteins, the presence of peptide signal and the number of α-helices. For each contig annotated as OBP or CSP, we indicated in which comparison both DESeq2 and EdgeR found it to be differentially expressed. D = domiciliary, P = peridomiciliary and S = sylvatic; M = male and F = female. The log2fold and the adjusted p-value for both packages are provided, as well as the cluster number allocated by HTSCluster. (DOCX) [file pntd.0005067.s003.docx]

| Name | Contig | Blast NR ID | Annotation | %ident | Score | E-value | Cysteins | Psignal | α Helice | DE in comparison | Log2fold | | Padj | | HTSCluster |
| --- | --- | --- | --- | --- | --- | --- | --- | --- | --- | --- | --- | --- | --- | --- | --- |
|  |  |  |  |  |  |  |  |  |  |  | DESeq2 | EdgeR | DESeq2 | EdgeR |  |
| TbraCSP1 | Contig8299 | gi\|349592318 | chemosensory protein 3 [Apolygus lucorum] | 59.52 | 163 | 3.00E-45 | 4 | 19 and 20 | 8 | D/P | -4.066 | -5.629 | 0.001 | 0.004 | 10 |
| TbraCSP2 | Contig13506 | gi\|349592314 | chemosensory protein 1 [Apolygus lucorum] | 62.07 | 133 | 8.00E-35 | 4 | 18 and 19 | 8 |  |  |  |  |  | 11 (P<0.9) |
| TbraCSP3 | Contig1318 | gi\|349592318 | chemosensory protein 3 [Apolygus lucorum] | 67.24 | 167 | 7.00E-45 | 4 | 20 and 21 | 8 | D/P | -3.186 | -3.761 | 0.003 | 0.023 | 22 |
| TbraCSP4 | Contig11307 | gi\|443908529 | chemosensory protein 8 [Apolygus lucorum] | 64.96 | 160 | 7.00E-47 | 4 | 18 and 19 | 8 | D/P | -3.190 | -3.929 | 0.004 | 0.007 | 19 |
| TbraCSP5 | Contig16746 | gi\|443908529 | chemosensory protein 8 [Apolygus lucorum] | 57.94 | 142 | 5.00E-38 | 4 | 18 and 19 | 8 |  |  |  |  |  | filtered |
| TbraCSP6 | Contig5540 | gi\|554894806 | chemosensory protein 6 [Laodelphax striatella] | 55.66 | 140 | 1.00E-35 | 4 | 18 and 19 | 8 | D/P | -3.247 | -4.828 | 0.013 | 0.019 | 19 |
| TbraCSP7 | Contig15930 | gi\|205326627 | chemosensory protein [Apis cerana cerana] | 65.05 | 142 | 2.00E-36 | 4 | 23 and 24 | 6 | D/P | -2.571 | -4.993 | 0.039 | 0.010 | 8 |
| TbraCSP8 | Contig13314 | gi\|215254086 | putative chemosensory protein CSP9 [Nilaparvata lugens] | 40.38 | 97.8 | 1.00E-20 | 4 | 22 and 23 | 8 |  |  |  |  |  | 8 (P<0.9) |
| TbraCSP9 | Contig4898 | gi\|669092314 | chemosensory proteins [Dendrolimus kikuchii] | 38.79 | 85.5 | 9.00E-17 | 4 | 22 and 23 | 7 | D/P | -3.060 | -3.952 | 0.012 | 0.007 | 10 |
| TbraCSP10 | Contig5394 | gi\|270000350 | chemosensory protein 4 [Adelphocoris lineolatus] | 46.6 | 106 | 5.00E-25 | 4 | 22 and 23 | 8 | D/P | -3.535 | -4.801 | 0.004 | 0.007 | 12 |
| TbraCSP11 | Supercontig_454_29433 | gi\|270000350 | chemosensory protein 4 [.] | 56.69 | 156 | 4.00E-42 | 4 | 18 and 19 | 8 | D/P | -3.521 | -4.719 | 0.004 | 0.014 | 2 |
| TbraCSP12 | Contig3848 | gi\|349592318 | chemosensory protein 3 [Apolygus lucorum] | 59.68 | 165 | 7.00E-46 | 4 | 20 and 21 | 8 | D/P | -2.945 | -3.802 | 0.014 | 0.033 | 2 |
| TbraCSP13 | Contig18893 | gi\|349592314 | chemosensory protein 1 [Apolygus lucorum] | 66.35 | 153 | 7.00E-44 | 4 | 18 and 19 | 8 | D/P | -2.556 | -3.132 | 0.019 | 0.028 | 16 |
| TbraCSP14 | Contig9262 | gi\|349592318 | chemosensory protein 3 [Apolygus lucorum] | 57.26 | 152 | 8.00E-44 | 4 | 18 and 19 | 8 | D/P | -3.027 | -5.409 | 0.020 | 0.004 | 1 |
|  |  |  |  |  |  |  |  |  |  | M/F | 3.803 | 5.707 | 1.849E-07 | 6.822E-06 |  |
| TbraCSP15 | comp176536 | gi\|349592318 | chemosensory protein 3 [Apolygus lucorum] | 60 | 131 | 1.00E-34 | 4 | 18 and 19 | 8 | D/P | -2.483 | -3.707 | 0.032 | 0.018 | filtered |
|  |  |  |  |  |  |  |  |  |  | M/F | 2.988 | 4.636 | 3.916E-04 | 0.005 |  |
| TbraCSP16 | Contig2436 | gi\|443908519 | chemosensory protein 3 [Apolygus lucorum] | 80 | 193 | 5.00E-56 | 4 | NO | 6 | D/P | -3.544 | -5.148 | 0.004 | 0.005 | 6 |
|  |  |  |  |  |  |  |  |  |  | M/F | 3.019 | 4.051 | 1.028E-05 | 9.745E-04 |  |
| TbraOBP1 | Contig6152 | gi\|33518701 | odorant-binding protein precursor [Rhodnius prolixus] | 80.14 | 244 | 3.00E-78 | 6 | 19 and 20 | 9 | D/P | -3.692 | -4.642 | 0.001 | 0.010 | 12 |
| TbraOBP2 | Contig6523 | gi\|23320749 | odorant-binding protein 2 precursor [Zootermopsis nevadensis] | 30 | 47 | 0.003 | 6 | 22 and 23 | 7 | D/P | -2.966 | -3.925 | 0.014 | 0.009 | 12 (P<0.9) |
| TbraOBP3 | Contig6047 | gi\|573006064 | odorant-binding protein 29 [Lygus lineolaris] | 55.49 | 188 | 1.00E-55 | 6 | 23 and 24 | 9 | D/P | -2.740 | -4.003 | 0.022 | 0.019 | 8 |
| TbraOBP4 | Contig18371 | gi\|573006060 | odorant-binding protein 27. partial [Lygus lineolaris] | 70.59 | 254 | 3.00E-79 | 6 | 25 and 26 | 8 | D/P | -2.796 | -4.034 | 0.022 | 0.006 | 8 |
|  |  |  |  |  |  |  |  |  |  | M/F | 2.058 | 2.828 | 0.045 | 0.009 |  |
| TbraOBP5 | comp179551_c0 | gi\|240247225 | odorant-binding protein RproOBP6 precursor [Rhodnius prolixus] | 90.34 | 280 | 1.00E-88 | 6 | 19 and 20 | 7 |  |  |  |  |  | 19 (P<0.9) |
| TbraOBP6 | comp181497_c0 | gi\|573006002 | odorant-binding protein 2 [Lygus lineolaris] | 63.95 | 194 | 2.00E-58 | 6 | 26 and 27 | 8 | D/P | -3.999 | -5.373 | 0.001 | 0.008 | 17 |
| TbraOBP7 | Contig17590 | gi\|240247187 | odorant-binding protein 13, partial [Acyrthosiphon pisum] | 47.73 | 92 | 4.00E-19 | 6 | NO | 5 | D/P | -2.811 | -3.524 | 0.018 | 0.011 | 24 |
| TbraOBP8 | Contig14874 | gi\|700284171 | putative odorant-binding protein 4 [Chinavia ubica] | 52 | 43.1 | 0.028 | 4 | 19 and 20 | 8 |  |  |  |  |  | 2 |
| TbraOBP9 | Contig11714 | gi\|291195901 | odorant-binding protein 1 [Apolygus lucorum] | 26.09 | 58.5 | 2.00E-07 | 6 | 22 and 23 | 7 | D/P | -2.495 | -3.523 | 0.035 | 0.015 | 10 |
| TbraOBP10 | Supercontig_454_840 | gi\|586830420 | Odorant-binding protein 8 [Adelphocoris suturalis] | 30.28 | 67.8 | 4.00E-10 | 4 | 21 and 22 | 7 |  |  |  |  |  | 20 (P<0.9) |
| TbraOBP11 | Contig11713 | gi\|573006042 | odorant-binding protein 19 [Lygus lineolaris] | 28.57 | 64.7 | 8.00E-10 | 4 | 21 and 22 | 8 | D/P | -3.593 | -4.674 | 0.002 | 0.004 | 12 |
| TbraOBP12 | Contig16927 | gi\|349592326 | Odorant-binding protein 4 [Apolygus lucorum] | 31 | 54.7 | 8.00E-06 | 4 | NO | 6 |  |  |  |  |  | filtered |
| TbraOBP13 | Contig11329 | gi\|240247221 | odorant-binding protein RproOBP4 precursor [Rhodnius prolixus] | 44.22 | 128 | 3.00E-33 | 6 | 18 and 19 | 7 | D/P | -4.390 | -5.885 | 2.22E-04 | 0.002 | 16 |
|  |  |  |  |  |  |  |  |  |  | D/S | -3.941 | -4.774 | 2.38E-06 | 0.009 |  |
| TbraOBP14 | comp178106_c0 | gi\|240247221 | odorant-binding protein RproOBP4 precursor [Rhodnius prolixus] | 66.44 | 185 | 6.00E-55 | 6 | 17 and 18 | 8 | D/P | -3.705 | -4.976 | 0.003 | 0.001 |  |
|  |  |  |  |  |  |  |  |  |  | D/S | -3.596 | -4.359 | 4.40E-05 | 0.002 | 16 |
| TbraOBP15 | Contig16558 | gi\|270000366 | odorant-binding protein 4 [Adelphocoris lineolatus] | 51.91 | 142 | 5.00E-37 | 6 | 22 and 23 | 7 |  |  |  |  |  | 15 |
| TbraOBP16 | Supercontig_454_5612 | gi\|270000366 | odorant-binding protein 4 [Adelphocoris lineolatus] | 51.91 | 142 | 5.00E-38 | 6 | 22 and 23 | 3 | D/P | -2.329 | -3.027 | 0.039 | 0.012 | 21 |
| TbraOBP17 | Contig16870 | gi\|700284175 | putative odorant-binding protein 1 [Telenomus podisi] | 56.34 | 87.8 | 5.00E-18 | 3 | NO | 5 |  |  |  |  |  | 24 (P<0.9) |
| TbraOBP18 | Contig3641 | gi\|270000360 | odorant-binding protein 1 [Adelphocoris lineolatus] | 45.45 | 135 | 5.00E-36 | 6 | 20 and 21 | 6 | D/P | -3.492 | -4.584 | 0.003 | 0.013 | 17 |
| TbraOBP19 | Contig14074 | gi\|240247219 | odorant-binding protein RproOBP2 precursor, partial [Rhodnius prolixus] | 29.06 | 55.1 | 9E-07 | 4 | NO | 7 |  |  |  |  |  | 6 |
| TbraOBP20 | Contig19415 | gi\|270000360 | odorant-binding protein 1 [Adelphocoris lineolatus] | 40.56 | 130 | 3E-33 | 6 | 19 and 20 | 7 | D/P | -3.643 | -4.531 | 0.002 | 0.011 | 2 |
| TbraOBP21 | Contig13477 | gi\|573459726 | putative odorant binding protein 77 [Nasonia vitripennis] | 32 | 43.1 | 0.033 | 6 | 30 and 31 | 6 | D/P | -3.605 | -4.593 | 0.002 | 0.006 | 12 |
| TbraOBP22 | Contig9624 | gi\|240247219 | odorant-binding protein RproOBP2 precursor. partial [Rhodnius prolixus] | 34.19 | 85.1 | 1.00E-16 | 6 | 19 and 20 | 7 | D/P | -4.239 | -5.278 | 1.85E-4 | 0.001 |  |
|  |  |  |  |  |  |  |  |  |  | D/S | -3.408 | -3.944 | 1.57E-05 | 0.019 | 14 |
| TbraOBP23 | Contig14361 | gi\|270000474 | odorant binding protein 12 [Adelphocoris lineolatus] | 59.57 | 66.6 | 3.00E-10 | 3 | 21 and 22 | 3 | D/P | -2.779 | -4.325 | 0.024 | 0.006 | 10 (P<0.9) |
| TbraOBP24 | Contig16524 | gi\|573006006 | odorant-binding protein 4 [Lygus lineolaris] | 55.93 | 160 | 1.00E-43 | 6 | 21 and 22 | 6 | D/P | -3.191 | -4.640 | 0.012 | 0.023 | 19 |
| TbraOBP25 | Contig12789 | gi\|240247219 | odorant-binding protein RproOBP2 precursor, partial [Rhodnius prolixus] | 30.77 | 68.9 | 3.00E-11 | 4 | 19 and 20 | 9 |  |  |  |  |  | 2 |

**Supplementary Table S1**: **Blast annotation of CSPs and OBPs, and information about differential expression status**.

Results of Blast: For all contigs that matched with a CSP or an OBP from the non-redundant protein database, we indicate the ID of this protein (Blast NR ID) and its denomination (Annotation). For each match, we provide the percentage of identity (%ident), the score and the e-value. Each contig that matches with an OBP or CSP was translated and the corresponding protein analyzed by checking for the number of conserved cysteins, the presence of peptide signal and the number of α-helices. For each contig annotated as OBP or CSP, we indicated in which comparison both DESeq2 and EdgeR found it to be differentially expressed. D = domiciliary, P = peridomiciliary and S = sylvatic; M = male and F = female. The log2fold and the adjusted *p*-value for both packages are provided, as well as the cluster number allocated by HTSCluster.
