# Supplementary material for: Under-Expression of Chemosensory Genes in Domiciliary Bugs of the Chagas Disease Vector Triatoma brasiliensis
Source: PLoS Negl Trop Dis. 2016 Oct 28;10(10):e0005067. doi: 10.1371/journal.pntd.0005067 (PMC5085048; doi:10.1371/journal.pntd.0005067)
Supplement: S2 Table — Results of Blast: For all contigs that matched with a protein of interest in the non-redundant protein database, we indicate the protein ID (Blast NR ID) and its denomination (Annotation). For each match, we provide the percentage of identity (%ident), the score and the e-value. For each contig, we indicate in which comparison both DESeq2 and EdgeR found it to be differentially expressed. D = domiciliary, P = peridomiciliary and S = sylvatic; M = male and F = female. The log2fold and the adjusted p-value for both packages are provided, as well as the cluster number allocated by HTSCluster. (DOCX) [file pntd.0005067.s004.docx]

| Contig | NR | Annotation | %id | Score | E-value | DE in comparison | Log2fold | | Padj | | HTS  Cluster |
| --- | --- | --- | --- | --- | --- | --- | --- | --- | --- | --- | --- |
|  |  |  |  |  |  |  | DESeq2 | EdgeR | DESeq2 | EdgeR |  |
| comp175751_c3 | gi\|703624121 | odorant receptor coreceptor [Cimex lectularius] | 831 | 89 | 0.0 |  |  |  |  |  | 18 |
| comp148596_c0 | gi\|270002959 | odorant receptor 32 [Tribolium castaneum] | 24 | 75.9 | 2.00E-12 |  |  |  |  |  | 19  (P<0.9) |
| comp175082_c0 | gi\|270006492 | odorant receptor 47 [Tribolium castaneum] | 27 | 65.5 | 3.00E -08 |  |  |  |  |  | 24  (P<0.9) |
| comp168189_c0 | gi\|604784347 | putative transient receptor potential cation channel protein painless-like isoform 1, partial [Triatoma infestans] | 99.16 | 1583 | 0.0 | D/P | -3.453 | -5.599 | 0.012 | 0.006 | 19 |
| Contig1540 | gi\|939258328 | PREDICTED: transient receptor potential channel pyrexia-like [Cimex lectularius] | 57.8 | 688 | 0.0 | D/P | -2.157 | -2.694 | 0.038 | 0.033 | 19 |
| comp164577_c1 | gi\|604782728 | putative transient receptor potential channel pyrexia-like protein, partial [Triatoma infestans] | 100 | 643 | 0.0 |  |  |  |  |  | 24  (P<0.9) |
| comp173354_c2 | gi\|604787447 | putative transient receptor potential cation channel cg34123-like protein, partial [Triatoma infestans] | 99.9 | 1548 | 0.0 |  |  |  |  |  | 23  (P<0.9) |
| Contig4472 | gi\|604782728 | putative transient receptor potential channel pyrexia-like protein, partial [Triatoma infestans] | 100.00 | 278 | 1.00E -85 |  |  |  |  |  | 8  (P<0.9) |
| comp168627_c0 | gi\|939262434 | PREDICTED: sensory neuron membrane protein 1-like [Cimex lectularius] | 69.49 | 774 | 0.0 | D/P | -3.453 | -5.599 | 0.012 | 0.006 | 17 |
| comp171165_c2 | gi\|328717644 | PREDICTED: protein takeout-like [Acyrthosiphon pisum] | 36.5 | 181 | 2.00E-49 | M/F | 2.874 | 3.948 | 6.01E-04 | 5.18E-04 | 5 |
| Contig9476 | gi\|389610927 | takeout/JHBP like protein [Papilio polytes] | 50.4 | 242 | 1.00E-73 | D/P | -2.607 | -4.010 | 0.027 | 0.010 | 9 |
|  |  |  |  |  |  | M/F | 2.290 | 3.324 | 0.011 | 0.006 |  |
| Contig3858 | gi\|662206712 | PREDICTED: protein takeout-like [Diaphorina citri] | 69.5 | 174 | 3.00E-70 |  |  |  |  |  | 9 |
| Contig16387 | gi\|662206712 | PREDICTED: protein takeout-like [Diaphorina citri] | 43.1 | 209 | 2.00E-61 | D/P | -3.334 | -4.845 | 0.008 | 0.006 | 2 |
|  |  |  |  |  |  | M/F | 4.058 | 5.182 | 2.77E-11 | 1.68E-05 |  |
| Contig11061 | gi\|328717644 | PREDICTED: protein takeout-like [Acyrthosiphon pisum] | 37.3 | 181 | 1.00E-49 | D/P | -2.929 | -5.754 | 0.025 | 0.015 | 19 |
| Contig242 | gi\|646720336 | Protein takeout [Zootermopsis nevadensis] | 39.8 | 207 | 1.00E-58 | D/P | -2.960 | -4.548 | 0.020 | 0.016 | 12 |
| Contig4693 | gi\|646707272 | Protein takeout [Zootermopsis nevadensis] | 56.1 | 294 | 2.00E-92 | D/P | -2.678 | -4.487 | 0.023 | 0.031 | 8 |
| Contig8939 | gi\|193645781 | PREDICTED: protein takeout-like [Acyrthosiphon pisum] | 42 | 192 | 2.00E-54 | D/P | -4.047 | -5.422 | 9.34E-04 | 0.007 | 12 |
| Contig15222 | gi\|326381129 | takeout-like [Acyrthosiphon pisum] | 40.8 | 211 | 2.00E-62 | D/P | -3.940 | -5.669 | 0.002 | 0.004 | 10 |
| Contig12334 | gi\|662206712 | PREDICTED: protein takeout-like [Diaphorina citri] | 38.4 | 155 | 2.00E-41 | D/P | -2.954 | -4.061 | 0.016 | 0.010 | 12 |
| comp158409_c0 | gi\|242022243 | protein takeout precursor, putative [Pediculus humanus corporis] | 30.1 | 79.7 | 2.00E-14 | D/P | -2.758 | -3.686 | 0.019 | 0.044 | 2 |
| Contig1601 | gi\|242024397 | protein takeout precursor, putative [Pediculus humanus corporis] | 37.02 | 160 | 4.00E -41 | D/P | -2.657 | -4.261 | 0.029 | 0.019 | 19 |
| Contig6740 | gi\|328717644 | PREDICTED: protein takeout-like [Acyrthosiphon pisum] | 43.33 | 214 | 4.00E -61 | D/P | -2.981 | -4.965 | 0.020 | 0.025 | 17 |
| comp171070_c0 | gi\|193636528 | PREDICTED: protein takeout-like [Acyrthosiphon pisum] | 48.46 | 237 | 3.00E-66 |  |  |  |  |  | 21 |
| Contig11584 | gi\|193636528 | PREDICTED: protein takeout-like [Acyrthosiphon pisum] | 41.38 | 211 | 2.00E-62 |  |  |  |  |  | 7 |
| Supercontig_454_5739 | gi\|193636528 | PREDICTED: protein takeout-like [Acyrthosiphon pisum] | 48.46 | 237 | 5.00E-69 |  |  |  |  |  | 15 |
| Contig17773 | gi\|242009643 | protein takeout precursor, putative [Pediculus humanus corporis] | 63.01 | 102 | 7.00E-24 |  |  |  |  |  | 21 (P<0.9) |
| comp158409_c0 | gi\|242022243 | protein takeout precursor, putative [Pediculus humanus corporis] | 30.08 | 79.7 | 2.00E-14 | D/P | -2.758 | -3.686 | 0.019 | 0.044 | 2 |
| Contig2664 | gi\|193636528 | PREDICTED: protein takeout-like [Acyrthosiphon pisum] | 44.22 | 239 | 2.00E-70 |  |  |  |  |  | 9 |
| mira_454_rep_c72681 | gi\|18140056 | takeout [Aedes aegypti] | 43.55 | 117 | 2.00E-27 | D/P | 2.980 | 8.346 | 0.030 | 0.004 | 17 (P<0.9) |
| comp126832_c0 | gi\|662206712 | PREDICTED: protein takeout-like [Diaphorina citri] | 51.54 | 246 | 3.00E-77 |  |  |  |  |  | 23 |
| Contig645 | gi\|662206712 | PREDICTED: protein takeout-like [Diaphorina citri] | 38.31 | 187 | 5.00E-52 | D/P | 2.757 | 3.346 | 0.015 | 0.045 | 16 |
| Contig6143 | gi\|326381129 | takeout-like [Acyrthosiphon pisum] | 39.58 | 112 | 1.00E-38 |  |  |  |  |  | 12 |
| Supercontig_454_2845 | gi\|339765128 | cytochrome P450 CYP417A1 [Nilaparvata lugens] | 30.5 | 113 | 4.00E-25 |  |  |  |  |  | 6 |
| comp174812_c0 | gi\|408724221 | cytochrome P450 CYP417A2v2 [Laodelphax striatella] | 29.9 | 179 | 7.00E-47 |  |  |  |  |  | 8 |
| comp145626_c1 | gi\|501295299 | cytochromeP450 [Riptortus pedestris] | 36.1 | 311 | 2.00E-96 |  |  |  |  |  | 6 |
| Supercontig_454_10385 | gi\|568259601 | cytochrome P450 [Anopheles darlingi] | 36.9 | 73.6 | 4.00E-15 |  |  |  |  |  | 12 (P<0.9) |
| comp171414_c0 | gi\|408724221 | cytochrome P450 CYP417A2v2 [Laodelphax striatella] | 29.3 | 203 | 4.00E-55 | M/F | 2.808 | 3.412 | 4.42E-05 | 9.00E-05 | 2 |
| comp154816_c0 | gi\|339896273 | cytochrome P450 [Bemisia tabaci] | 50 | 191 | 1.00E-53 |  |  |  |  |  | 23 (P<0.9) |
| Contig11448 | gi\|501295299 | cytochromeP450 [Riptortus pedestris] | 44.1 | 194 | 2.00E-52 | D/P | -2.757 | -3.346 | 0.015 | 0.045 | 22 (P<0.9) |
| Supercontig_454_43924 | gi\|391346350 | PREDICTED: cytochrome P450 3A31-like [Metaseiulus occidentalis] | 32.7 | 92.4 | 1.00E-16 |  |  |  |  |  | 3 |
| comp146998_c0 | gi\|31223075 | cytochrome P450 CYP6Y1 [Anopheles gambiae] | 39.3 | 225 | 1.00E-65 |  |  |  |  |  | 12 (P<0.9) |
| Supercontig_454_1667 | gi\|564227928 | PREDICTED: cytochrome P450 4V2-like, partial [Alligator mississippiensis] | 52.1 | 211 | 3.00E-63 | D/P | -2.561 | -3.304 | 0.020 | 0.011 | 17 |
| Contig19271 | gi\|501295299 | cytochromeP450 [Riptortus pedestris] | 43.9 | 135 | 4.00E-45 | D/P | -2.756 | -3.586 | 0.021 | 0.005 | 10 (P<0.9) |
| Contig8480 | gi\|501295299 | cytochromeP450 [Riptortus pedestris] | 44.4 | 478 | 7.00E-159 | D/P | -2.666 | -3.472 | 0.022 | 0.028 | 17 |
| Contig4340 | gi\|408724221 | cytochrome P450 CYP417A2v2 [Laodelphax striatella] | 40.2 | 179 | 6.00E-47 | D/P | -2.953 | -4.233 | 0.017 | 0.011 | 19 |
| Contig18531 | gi\|646701016 | Cytochrome P450 9e2 [Zootermopsis nevadensis] | 38.7 | 340 | 1.00E-102 | D/P | -2.568 | -3.306 | 0.022 | 0.024 | 17 |
| Contig1415 | gi\|501295299 | cytochromeP450 [Riptortus pedestris] | 38.4 | 322 | 3.00E-98 | D/P | -2.926 | -4.367 | 0.019 | 0.015 | 17 |
| newbler_454_04299 | gi\|510813177 | cytochrome P450, partial [Laodelphax striatella] | 48.4 | 139 | 8.00E-35 | D/P | -2.532 | -3.613 | 0.030 | 0.015 | 17 |
| comp174285_c0 | gi\|646701016 | Cytochrome P450 9e2 [Zootermopsis nevadensis] | 36 | 290 | 4.00E-87 | D/P | -3.130 | -4.069 | 0.010 | 0.004 | 19 |
| Supercontig_454_7993 | gi\|408724221 | cytochrome P450 CYP417A2v2 [Laodelphax striatella] | 38.8 | 205 | 2.00E-57 | D/P | -3.743 | -4.849 | 0.002 | 0.007 | 2 |
|  |  |  |  |  |  | D/S | -3.635 | -4.266 | 6.87E-06 | 0.045 |  |
| Contig2856 | gi\|646716643 | putative cytochrome P450 6a13 [Zootermopsis nevadensis] | 31.4 | 177 | 4.00E-80 | D/P | -3.545 | -5.407 | 0.009 | 0.006 | 12 |
| Contig9883 | gi\|408724221 | cytochrome P450 CYP417A2v2 [Laodelphax striatella] | 39.2 | 212 | 4.00E-59 | D/P | -3.743 | -4.849 | 0.002 | 0.007 | 2 |
|  |  |  |  |  |  | D/S | -3.478 | -4.069 | 3.06E-05 | 0.043 |  |
| comp175484_c9 | gi\|501295299 | cytochromeP450 [Riptortus pedestris] | 47.4 | 154 | 1.00E-39 | D/P | -3.774 | -5.112 | 0.002 | 0.005 | 16 |
|  |  |  |  |  |  | D/S | -4.052 | -4.896 | 2,60E-07 | 0.009 |  |
| Contig18029 | gi\|270006370 | cytochrome P450 6BQ4 [Tribolium castaneum] | 32.4 | 161 | 2.00E-64 | D/P | -3.856 | -5.108 | 0.002 | 0.007 | 17 |
| Contig13659 | gi\|408724221 | cytochrome P450 CYP417A2v2 [Laodelphax striatella] | 35 | 211 | 1.00E-58 | D/P | -3.648 | -6.102 | 0.008 | 0.004 | 12 |
| mira_454_rep_c18806 | gi\|408724221 | cytochrome P450 CYP417A2v2 [Laodelphax striatella] | 47.5 | 179 | 3.00E-48 | D/P | -2.638 | -3.276 | 0.021 | 0.006 | 21 (P<0.9) |
| Contig2511 | gi\|501295299 | cytochromeP450 [Riptortus pedestris] | 35.7 | 359 | 5.00E-112 | D/P | -3.087 | -5.167 | 0.020 | 0.013 | 2 |
| comp172878_c4 | gi\|645004806 | PREDICTED: cytochrome P450 4g15 [Nasonia vitripennis] | 31.4 | 95.1 | 3.00E-18 | D/P | -3.172 | -4.229 | 0.014 | 0.008 | 16 (P<0.9) |
|  |  |  |  |  |  | D/S | -3.249 | -3.896 | 6.62E-04 | 0.043 |  |
| comp175484_c3 | gi\|501295299 | cytochromeP450 [Riptortus pedestris] | 38 | 193 | 3.00E-49 | D/P | -3.021 | -4.084 | 0.017 | 0.019 | 21 |
| comp120212_c0 | gi\|646707025 | putative cytochrome P450 CYP44 [Zootermopsis nevadensis] | 40.21 | 398 | 2.00E-129 | M/F | 1.918 | 2.235 | 0.023 | 0.010 | 1 |
| comp126058_c1 | gi\|61611906 | cytochrome P450 [Culex pipiens pallens] | 34.28 | 315 | 8.00E-97 | D/P | -2.597 | -3.625 | 0.028 | 0.008 | 24 |
| comp147212_c0 | gi\|662213714 | PREDICTED: cytochrome P450 302a1, mitochondrial [Diaphorina citri] | 45.79 | 199 | 4.00E-56 |  |  |  |  |  | 6 (P<0.9) |
| comp147602_c0 | gi\|60686423 | cytochrome P450 [Mayetiola destructor] | 33.85 | 126 | 5.00E-30 |  |  |  |  |  | 15 (P<0.9) |
| comp150255_c0 | gi\|501295299 | cytochromeP450 [Riptortus pedestris] | 34.62 | 198 | 5.00E-55 | D/P | -2.717 | -3.675 | 0.025 | 0.010 | 12 |
| comp163607_c0 | gi\|291464089 | cytochrome P450 4G4 [Manduca sexta] | 65.91 | 732 | 0.0 | D/P | -2.605 | -3.252 | 0.020 | 0.014 | 17 |
| comp163933_c1 | gi\|498928672 | PREDICTED: cytochrome P450 4g1-like [Ceratitis capitata] | 48.37 | 149 | 4.00E-37 | D/P | -2.231 | -2.928 | 0.048 | 0.019 | 21 (P<0.9) |
| comp165506_c0 | gi\|408724249 | cytochrome P450 CYP18A1, partial [Laodelphax striatella] | 69.88 | 364 | 6.00E-121 |  |  |  |  |  | 15 (P<0.9) |
| comp168063_c0 | gi\|380015389 | PREDICTED: cytochrome P450 306a1-like, partial [Apis florea] | 38.95 | 148 | 2.00E-38 |  |  |  |  |  | 18 (P<0.9) |
| comp168551_c0 | gi\|501295299 | cytochromeP450 [Riptortus pedestris] | 40.05 | 345 | 7.00E-110 | D/P | -2.833 | -3.537 | 0.015 | 0.008 | 21 (P<0.9) |
| comp170482_c1 | gi\|501295299 | cytochromeP450 [Riptortus pedestris] | 42.89 | 364 | 4.00E-117 | D/P | - 2.314 | -2.858 | 0.030 | 0.018 | 17 (P<0.9) |
| comp173258_c1 | gi\|383857845 | PREDICTED: probable cytochrome P450 6a14-like [Megachile rotundata] | 42.01 | 188 | 3.00E-52 |  |  |  |  |  | 11 (P<0.9) |
| comp173258_c2 | gi\|478734979 | cytochrome P450 CYP6BQ22 [Dastarcus helophoroides] | 47.01 | 238 | 1.00E-69 |  |  |  |  |  | 19 |
| comp173921_c0 | gi\|408724329 | cytochrome P450 CYP6AY3v2 [Laodelphax striatella] | 32.72 | 279 | 3.00E-80 |  |  |  |  |  | 17 (P<0.9) |
| comp174285_c0 | gi\|646701016 | Cytochrome P450 9e2 [Zootermopsis nevadensis] | 35.98 | 289 | 4.00E-87 | D/P | -3.130 | -4.069 | 0.011 | 0.004 | 19 |
| comp174572_c3 | gi\|646720986 | Cytochrome P450 4C1 [Zootermopsis nevadensis] | 32.97 | 93.2 | 1.00E-16 |  |  |  |  |  | 21 |
| comp175018_c6 | gi\|350397446 | PREDICTED: probable cytochrome P450 301a1, mitochondrial-like isoform 1 [Bombus impatiens] | 68.04 | 501 | 1.00E-170 |  |  |  |  |  | 8 |
| comp175049_c2 | gi\|339896295 | cytochrome P450 [Bemisia tabaci] | 50.00 | 63.5 | 3.00E-08 |  |  |  |  |  | 24 (P<0.9) |
| comp175685_c2 | gi\|408724221 | cytochrome P450 CYP417A2v2 [Laodelphax striatella] | 26.85 | 147 | 2.00E-34 |  |  |  |  |  | 2 |
| comp176576_c1 | gi\|408724329 | cytochrome P450 CYP6AY3v2 [Laodelphax striatella] | 51.00 | 513 | 1.00E-174 |  |  |  |  |  | 15 (P<0.9) |
| comp294098_c0 | gi\|646701016 | Cytochrome P450 9e2 [Zootermopsis nevadensis] | 40.24 | 330 | 1.00E-103 | D/P | -2.261 | -2.866 | 0.034 | 0.011 | 10 |
| comp55327_c0 | gi\|646701016 | Cytochrome P450 9e2 [Zootermopsis nevadensis] | 39.31 | 341 | 4.00E-107 | D/P | -2.584 | -3.387 | 0.026 | 0.015 | 12 |
| Contig10143 | gi\|501291494 | cytochromeP450 [Riptortus pedestris] | 54.48 | 166 | 4.00E-48 | D/P | -2.475 | -3.126 | 0.025 | 0.004 | 21 (P<0.9) |
| Contig10144 | gi\|646477539 | putative cytochrome P450 6a13 [Zootermopsis nevadensis] | 50.00 | 91.7 | 1.00E-20 |  |  |  |  |  | filtered |
| Contig10638 | gi\|242006280 | NADPH--cytochrome P450, putative [Pediculus humanus corporis] | 93.94 | 68.9 | 1.00E-10 |  |  |  |  |  | 15 (P<0.9) |
| Contig12459 | gi\|19879426 | cytochrome P450 monooxygenase CYP6X1v2 [Lygus lineolaris] | 48.17 | 355 | 2.00E-110 | D/P | -2.597 | -3.950 | 0.030 | 0.028 | 8 |
| Contig17074 | gi\|642938945 | PREDICTED: probable cytochrome P450 6a23, partial [Tribolium castaneum] | 45.77 | 142 | 1.00E-37 |  |  |  |  |  | 24 (P<0.9) |
| Contig18608 | gi\|408724221 | cytochrome P450 CYP417A2v2 [Laodelphax striatella] | 57.83 | 108 | 2.00E-23 | D/P | -2.651 | -3.485 | 0.020 | 0.019 | 21 |
| Contig18738 | gi\|408724221 | cytochrome P450 CYP417A2v2 [Laodelphax striatella] | 34.73 | 229 | 5.00E-65 | D/P | -2.534 | -3.2264 | 0.023 | 0.007 | 12 (P<0.9) |
| Contig2982 | gi\|380293835 | NADPH-cytochrome P450 reductase [Cimex lectularius] | 83.46 | 1171 | 0.0 |  |  |  |  |  | 2 |
| Contig3204 | gi\|646701016 | Cytochrome P450 9e2 [Zootermopsis nevadensis] | 37.89 | 381 | 2.00E-119 |  |  |  |  |  | 2 |
| Contig3760 | gi\|501295299 | cytochromeP450 [Riptortus pedestris] | 44.53 | 442 | 5.00E-146 | D/P | 2.891 | 3.646 | 0.013 | 0.034 | 21 |
| Contig3961 | gi\|383860255 | PREDICTED: cytochrome P450 6a2-like [Megachile rotundata] | 35.02 | 140 | 4.00E-34 |  |  |  |  |  | 18 (P<0.9) |
| Contig4442 | gi\|646701016 | Cytochrome P450 9e2 [Zootermopsis nevadensis] | 38.69 | 372 | 4.00E-114 |  |  |  |  |  | 19 |
| Contig6425 | gi\|408724255 | cytochrome P450 CYP6BD10v2 [Laodelphax striatella] | 42.34 | 361 | 1.00E-108 | D/P | -2.424 | -4.154 | 0.048 | 0.019 | 24 |
| Contig8480 | gi\|501295299 | cytochromeP450 [Riptortus pedestris] | 44.40 | 478 | 7.00E-159 | D/P | -2.666 | -3.472 | 0.022 | 0.028 | 17 |
| Contig9376 | gi\|501295299 | cytochromeP450 [Riptortus pedestris] | 40.17 | 260 | 7.00E-78 |  |  |  |  |  | 18 (P<0.9) |
| mira_454_rep_c4693 | gi\|662186779 | PREDICTED: cytochrome P450 4g1-like [Diaphorina citri] | 51.56 | 70.9 | 5.00E-12 |  |  |  |  |  | Filtered |
| newbler_454_02565 | gi\|91084715 | cytochrome P450 6BT1 [Tribolium castaneum] | 50.00 | 91.3 | 1.00E-17 |  |  |  |  |  | 15 (P<0.9) |
| Supercontig_454_24630 | gi\|391346350 | PREDICTED: cytochrome P450 3A31-like [Metaseiulus occidentalis] | 36.96 | 45.8 | 1.00E-06 |  |  |  |  |  | 20 (P<0.9) |
| Supercontig_454_43655 | gi\|646720986 | Cytochrome P450 4C1 [Zootermopsis nevadensis] | 36.90 | 58.9 | 4.00E-06 |  |  |  |  |  | 22 |
| Supercontig_454_46254 | gi\|339896301 | cytochrome P450 [Bemisia tabaci] | 39.34 | 149 | 5.00E-38 | D/P | -2.958 | -4.332 | 0.021 | 0.001 | 19 (P<0.9) |
| Supercontig_454_7517 | gi\|501295299 | cytochromeP450 [Riptortus pedestris] | 30.77 | 125 | 4.00E-29 |  |  |  |  |  | 16 (P<0.9) |
| Supercontig_454_829 | gi\|662186779 | PREDICTED: cytochrome P450 4g1-like [Diaphorina citri] | 51.56 | 70.9 | 5.00E-12 |  |  |  |  |  | Filtered |
| comp161828_c0 | gi\|387413531 | glutathione s-transferase T1 [Sogatella furcifera] | 54.1 | 248 | 3.00E-77 | D/P | -2.259 | -3.096 | 0.041 | 0.022 | 8 |
| Contig13312 | gi\|700894839 | glutathione S-transferase [Diaphorina citri] | 41.5 | 143 | 4.00E-38 | D/P | -2.364 | -3.574 | 0.042 | 0.017 | 8 |
| Contig14872 | gi\|387413743 | glutathione s-transferase Z1 [Sogatella furcifera] | 89.4 | 414 | 1.00E-142 | D/P | -2.892 | -4.450 | 0.020 | 0.014 | 19 |
| comp171728_c1 | gi\|641654330 | PREDICTED: glutathione S-transferase theta-1-like [Acyrthosiphon pisum] | 53.7 | 258 | 2.00E-81 |  |  |  |  |  | 21 |
| comp175562_c1 | gi\|387413424 | glutathione s-transferase M2 [Nilaparvata lugens] | 66.67 | 204 | 6.00E -60 |  |  |  |  |  | 18 |
| Supercontig_454_1650 | gi\|817068565 | PREDICTED: larval cuticle protein A2B-like [Athalia rosae] | 76.9 | 112 | 2.00E-26 | M/F | 3.402 | 4.184 | 1.79E-07 | 3.19E-04 | 2 |
| Contig15155 | gi\|646706208 | Larval cuticle protein A3A [Zootermopsis nevadensis] | 88.3 | 116 | 8.00E-28 | D/P | -3.131 | -4.999 | 0.017 | 0.005 | 2 |
|  |  |  |  |  |  | M/F | 3.330 | 4.704 | 7.78E-06 | 1.24E-04 |  |
| Supercontig_454_44553 | gi\|817068565 | PREDICTED: larval cuticle protein A2B-like [Athalia rosae] | 76.9 | 110 | 2.00E-25 | M/F | 2.402 | 2.921 | 0.003 | 0.002 | 6 |
| comp173141_c0 | gi\|117622 | Cuticle protein 8 [Locusta migratoria] | 81 | 107 | 9.00E-25 | M/F | 3.260 | 3.942 | 4.41E-06 | 1.24E-04 | 2 |
| Contig5048 | gi\|817214821 | PREDICTED: cuticle protein 21-like [Orussus abietinus] | 75 | 132 | 1.00E-34 | S/P | -3.040 | -4.400 | 0.035 | 0.003 | 1 |
|  |  |  |  |  |  | D/P | -2.208 | -3.652 | 0.045 | 0.017 |  |
|  |  |  |  |  |  | M/F | 5.100 | 6.435 | 9.62E-19 | 5.05E-09 |  |
| Supercontig_454_438 | gi\|817054670 | PREDICTED: cuticle protein 19-like [Athalia rosae] | 84.1 | 123 | 2.00E-29 | D/P | -2.864 | -5.149 | 0.023 | 0.019 | 8 |
|  |  |  |  |  |  | M/F | 2.964 | 4.544 | 2.86E-04 | 0.008 |  |
| Contig9755 | gi\|117622 | Cuticle protein 8 [Locusta migratoria] | 59.7 | 71.6 | 4.00E-11 | D/P | -3.141 | -4.013 | 0.007 | 0.009 | 17 |
|  |  |  |  |  |  | M/F | 1.905 | 2.384 | 0.028 | 0.034 |  |
| comp167139_c0 | gi\|501291136 | cuticle protein, putative [Riptortus pedestris] | 91.9 | 120 | 7.00E-29 | D/P | -3.359 | -5.050 | 0.011 | 0.004 | 2 |
|  |  |  |  |  |  | M/F | 3.319 | 4.558 | 3.41E-06 | 1.26E-04 |  |
| Contig7919 | gi\|646704183 | Cuticle protein [Zootermopsis nevadensis] | 62.5 | 152 | 1.00E-38 | M/F | 2.360 | 4.180 | 0.031 | 0.016 | 5 |
| comp155262_c0 | gi\|646715957 | Cuticle protein 7 [Zootermopsis nevadensis] | 68.8 | 122 | 1.00E-28 |  |  |  |  |  | filtered |
| Contig1532 | gi\|240848893 | cuticular protein CPG12-like precursor [Acyrthosiphon pisum] | 82.3 | 87.8 | 7.00E-16 | D/P | -3.304 | -4.972 | 0.012 | 0.009 | 9 |
|  |  |  |  |  |  | M/F | 2.163 | 3.170 | 0.032 | 0.025 |  |
| Supercontig_454_5892 | gi\|646715957 | Cuticle protein 7 [Zootermopsis nevadensis] | 68.8 | 122 | 2.00E-28 | D/P | -3.395 | -6.019 | 0.012 | 0.003 | 5 |
|  |  |  |  |  |  | S/P | -3.688 | -5.693 | 0.009 | 2.50E-04 |  |
|  |  |  |  |  |  | M/F | 3.407 | 5.278 | 5.19E-06 | 8.23E-05 |  |
| Supercontig_454_5648 | gi\|166851824 | cuticular protein analogous to peritrophins 3-C5 isoform 2 precursor [Tribolium castaneum] | 81.8 | 411 | 1.00E-140 | D/P | -3.010 | -3.832 | 0.012 | 0.022 | 2 |
| Contig6804 | gi\|662225759 | PREDICTED: larval cuticle protein 1-like, partial [Diaphorina citri] | 77.3 | 130 | 6.00E-32 | D/P | -3.958 | -4.807 | 2.23E-04 | 0.003 | 14 |
| Contig11778 | gi\|389611489 | cuticular protein PxutCPR146, partial [Papilio xuthus] | 37.7 | 81.6 | 9.00E-14 | D/P | -2.873 | -3.795 | 0.016 | 0.018 | 19 |
| comp173496_c1 | gi\|357614984 | cuticular protein RR-2 motif 79 [Danaus plexippus] | 85.7 | 139 | 2.00E-31 | D/P | -2.432 | -3.413 | 0.0349 | 0.047 | 9 |
| Contig574 | gi\|646704638 | Endocuticle structural glycoprotein SgAbd-2, partial [Zootermopsis nevadensis] | 41.8 | 123 | 9.00E-31 | D/P | -2.858 | -3.754 | 0.016 | 0.014 | 10 |
| comp171408_c0 | gi\|759035757 | PREDICTED: pupal cuticle protein 20-like [Cerapachys biroi] | 51.2 | 99 | 2.00E-20 | D/P | -2.631 | -5.491 | 0.039 | 0.020 | 9 |
| Contig12583 | gi\|662197351 | PREDICTED: endocuticle structural glycoprotein SgAbd-2-like [Diaphorina citri] | 65.7 | 130 | 8.00E-33 |  |  |  |  |  | 4 |
| comp107934_c0 | gi\|290558788 | cuticular protein RR-1 motif 33 precursor [Bombyx mori] | 65.15 | 157 | 2.00E-43 |  |  |  |  |  | 4 |
| comp158087_c0 | gi\|665800845 | PREDICTED: adult-specific cuticular protein ACP-22-like [Microplitis demolitor] | 68.75 | 108 | 2.00E-24 |  |  |  |  |  | 20 (P<0.9) |
| comp170397_c0 | gi\|290560754 | cuticular protein RR-2 motif 73 precursor [Bombyx mori] | 89.19 | 75.9 | 2.00E-12 |  |  |  |  |  | 15 |
| comp178445_c0 | gi\|662184166 | PREDICTED: endocuticle structural glycoprotein SgAbd-9-like [Diaphorina citri] | 53.92 | 101 | 2.00E-22 |  |  |  |  |  | 9 |
| comp179689_c0 | gi\|157126722 | cuticle protein, putative [Aedes aegypti] | 75.36 | 123 | 6.00E-31 | M/F | 3.165 | 4.087 | 7.85E-06 | 3.64E-05 | 5 |
| Contig1370 | gi\|501291136 | cuticle protein, putative [Riptortus pedestris] | 84.72 | 130 | 8.00E-33 |  |  |  |  |  | 9 |
| Contig185 | gi\|646714048 | Larval cuticle protein 2 [Zootermopsis nevadensis] | 65.69 | 138 | 3.00E-34 |  |  |  |  |  | 9 |
| Contig2710 | gi\|157129506 | pupal cuticle protein, putative [Aedes aegypti] | 75.29 | 129 | 1.00E-33 | D/P | -2.827 | -3.821 | 0.016 | 0.007 | 8 |
| Contig6354 | gi\|501293980 | cuticle protein, putative [Riptortus pedestris] | 47.02 | 70.5 | 8.00E-10 |  |  |  |  |  | 4 |
| Contig6573 | gi\|350406207 | PREDICTED: endocuticle structural glycoprotein SgAbd-2-like [Bombus impatiens] | 65.79 | 144 | 4.00E-37 |  |  |  |  |  | 9 |
| Contig7304 | gi\|253735765 | cuticle protein precursor [Acyrthosiphon pisum] | 68.85 | 97.1 | 1.00E-21 |  |  |  |  |  | 9 (P<0.9) |
| Contig7887 | gi\|646704638 | Endocuticle structural glycoprotein SgAbd-2, partial [Zootermopsis nevadensis] | 54.81 | 127 | 3.00E-31 |  |  |  |  |  | 18 |
| mira_454_rep_c244 | gi\|642924550 | PREDICTED: endocuticle structural glycoprotein SgAbd-8 isoform X2 [Tribolium castaneum] | 70.69 | 147 | 1.00E-38 |  |  |  |  |  | filtered |
| Supercontig_454_1887 | gi\|642913097 | PREDICTED: LOW QUALITY PROTEIN: larval cuticle protein A2B-like [Tribolium castaneum] | 63.25 | 123 | 6.00E-31 | D/P | -2.545 | -3.756 | 0.033 | 0.011 | 2 |
|  |  |  |  |  |  | M/F | 2.983 | 3.801 | 5.80E-05 | 2.26E-04 |  |
| Supercontig_454_3182 | gi\|1706197 | RecName: Full=Pupal cuticle protein C1B; AltName: Full=TM-PCP C1B; Short=TM-C1B [Tenebrio molitor] | 78.18 | 87.4 | 1.00E-17 | M/F | 3.449 | 4.810 | 3.41E-06 | 6.92E-06 | 1 |
| Supercontig_454_33795 | gi\|253735765 | cuticle protein precursor [Acyrthosiphon pisum] | 68.85 | 96.3 | 6.00E-21 |  |  |  |  |  | 3 (P<0.9) |
| Supercontig_454_5996 | gi\|662197351 | PREDICTED: endocuticle structural glycoprotein SgAbd-2-like [Diaphorina citri] | 71.91 | 124 | 3.00E-31 |  |  |  |  |  | filtered |
| Supercontig_454_6096 | gi\|568250246 | pupal cuticle protein 78E [Anopheles darlingi] | 57.14 | 125 | 1.00E-31 | D/P | -2.746 | -4.719 | 0.029 | 0.017 | 9 |

**Supplementary Table S2**: **Blast annotation of ORs, TRPs, SNMPs, takeout, cytochrome P450, glutathione S-transferase and cuticular proteins and information about differential expression status.**

Results of Blast: For all contigs that matched with a protein of interest in the non-redundant protein database, we indicate the protein ID (Blast NR ID) and its denomination (Annotation). For each match, we provide the percentage of identity (%ident), the score and the e-value. For each contig, we indicate in which comparison both DESeq2 and EdgeR found it to be differentially expressed. D = domiciliary, P = peridomiciliary and S = sylvatic; M = male and F = female. The log2fold and the adjusted *p*-value for both packages are provided, as well as the cluster number allocated by HTSCluster.
